# Genetic Differentiation in the SdhC Subunit Confers Intrinsic Resistance to SDHI Fungicides in *Fusarium asiaticum*

**DOI:** 10.1111/mpp.70269

**Published:** 2026-05-05

**Authors:** Jichang Song, Jiakai Wang, Xudong Liu, Yinkai Liu, Lianyu Bi, Meixia Li, Yiqiang Cai, Mingguo Zhou, Yabing Duan

**Affiliations:** ^1^ State Key Laboratory of Agricultural and Forestry Biosecurity, College of Plant Protection Nanjing Agricultural University Nanjing Jiangsu China

**Keywords:** FaSdhC1, FaSdhC2, *Fusarium asiaticum*, resistance, SDHI fungicides

## Abstract

Fusarium head blight (FHB), caused by *Fusarium asiaticum*, is a significant wheat disease in China, reducing yields and contaminating grain with deoxynivalenol (DON). Due to the absence of highly resistant wheat varieties, chemical control remains the primary method for managing FHB. This study identified two homologous *SDHC* genes in *F. asiaticum*, namely *FaSDHC1* and *FaSDHC2*. Deletion of *FaSDHC2* impaired fungal sporulation, virulence and DON production, whereas the loss of *FaSDHC1* increased DON production. Both genes were found to regulate sensitivity to SDHI fungicides—ΔFaSDHC1 showed decreased sensitivity, while ΔFaSDHC2 exhibited increased sensitivity. To explore the regulatory role of the SdhC subunit in SDHI fungicides sensitivity in *F. asiaticum*, this study obtained 11 boscalid‐resistant mutants from ΔFaSDHC2 through chemical taming. Genetic analysis identified six mutation genotypes, including two previously reported in *Fusarium* species (FaSdhB‐H248Y and FaSdhD‐E166K) and four novel mutations in the *Fusarium* genus (FaSdhC1‐H144Y, FaSdhC1‐H144N, FaSdhD‐H122Y and FaSdhD‐D133N). The site‐directed mutation verification and sensitivity assays revealed that mutants harbouring FaSdhC1‐H144Y/N, FaSdhD‐H122Y, FaSdhD‐D133N or FaSdhD‐E166K exhibited resistance to multiple SDHI fungicides (boscalid, fluopyram, pydiflumetofen, isopyrazam and benzovindiflupyr). In contrast, FaSdhB‐H248Y mutants remained sensitive to fluopyram while showing resistance to other SDHI fungicides. This study elucidates the important regulatory role of SdhC genetic differentiation to SDHI fungicide sensitivity in *F. asiaticum*. Furthermore, it confirms that specific variations within distinct subunits of the SDH complex endow *F. asiaticum* with resistance to SDHI fungicides. The findings advance understanding of SDHI fungicides resistance mechanisms and provide a basis for developing FaSdhC‐specific inhibitors and resistance management strategies.

## Graphical abstract

In *Fusarium asiaticum*, two homologous SdhC subunits (FaSdhC1/C2) regulate sensitivity to SDHI fungicides: deletion of *FaSDHC1* decreases sensitivity, while deletion of *FaSDHC2* increases it. Multiple mutations in ΔFaSDHC2 confer resistance to SDHI fungicides. Combining a FaSdhC2‐specific inhibitor with SDHI fungicides may enhance control efficacy and decrease the resistance risk.

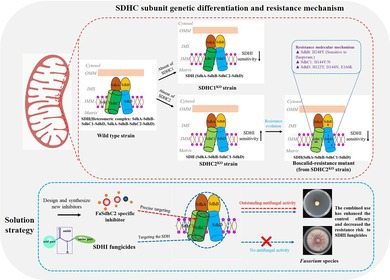

## 1 Introduction

Fusarium head blight (FHB), caused by the *Fusarium graminearum* species complex (FGSC), poses a significant threat to global cereal production, resulting in substantial reductions in yield (Goswami and Kistler 2004). In China, *Fusarium asiaticum* has been identified as the predominant fungal pathogen responsible for FHB in wheat crops (Qu et al. 2008; Glinka and Liao 2011). This pathogen is capable of producing several mycotoxins, particularly deoxynivalenol (DON), which significantly threatens grain safety and poses risks to human health (Goswami and Kistler 2004; Feizollahi et al. 2021). Therefore, effectively managing FHB is crucial for protecting agricultural economies and ensuring public health.

Currently, the lack of highly FHB‐resistant wheat varieties suitable for widespread cultivation necessitates the continued use of a chemical control strategy. However, the prolonged reliance on single fungicides—such as the benzimidazole fungicide carbendazim and the sterol demethylation inhibitors (DMIs) tebuconazole and prochloraz—has resulted in the emergence of resistant strains in the field (Duan et al. 2016; Duan, Tao, et al. 2019; Zhao et al. 2022). The increase in resistant populations has significantly diminished the effectiveness of these fungicides, highlighting the urgent need for the development of fungicides with novel modes of action.

In recent years, succinate dehydrogenase inhibitor (SDHI) fungicides have shown considerable potential in the fungicide market due to their unique mechanisms and effective control of various pathogens (Li et al. 2021). SDHI fungicides function by inhibiting the succinate dehydrogenase enzyme complex (SDH, Complex II) within the fungal mitochondrial respiratory chain. This inhibition disrupts their energy metabolism, thereby effectively suppressing fungal growth. These fungicides exhibit substantial efficacy against a diverse array of pathogens and are extensively used in the management of various crop diseases (Song, Qiu, et al. 2024; Duan, Xiu, et al. 2019; Bi et al. 2022). However, due to the structural differences among various SDHI fungicides, only a limited number are currently registered for the control of FHB. Research has demonstrated that certain SDHI fungicides can significantly inhibit both the mycelial growth and DON biosynthesis in *F. asiaticum* (Xu et al. 2019). This discovery presents new opportunities for integrated management strategies to combat FHB.

The SDH enzyme complex consists of four core subunits: SdhA, SdhB, SdhC and SdhD. SdhA and SdhB are soluble proteins situated within the mitochondrial matrix, while SdhC and SdhD are membrane‐bound subunits embedded in the inner mitochondrial membrane (Sierotzki and Scalliet 2013). Research has shown that amino acid mutations at specific sites within the SdhB, SdhC and SdhD subunits constitute the principal molecular mechanisms that confer resistance to SDHI fungicides in fungal pathogens (Leach and Lindow 2018). Notably, in numerous plant‐pathogenic fungi, the SdhC subunit is not encoded by a single gene; instead, it exhibits functional differentiation. For instance, in *Zymoseptoria tritici*, SdhC is differentiated into SdhC1, SdhC2 and SdhC3 (Steinhauer et al. 2020), while in *Fusarium* species, it is distinguished into SdhC1 and SdhC2 (Chen et al. 2024; Li et al. 2025). This differentiation implies that these paralogs may serve distinct functions in fungal biology. A comprehensive examination of this functional divergence is essential for enhancing the efficacy of SDHI fungicides and developing effective resistance management strategies.

A comprehensive understanding of fungicide resistance mechanisms is essential for monitoring resistance development, establishing effective management strategies and optimizing new fungicide formulations. Given the distinct functions of the SdhC subunits and their crucial role in the evolution of resistance, it has become vital to elucidate the specific roles of FaSdhC1 and FaSdhC2 in *F. asiaticum* to comprehend SDHI fungicide resistance and devise innovative strategies. In this study, we systematically compared the roles of FaSdhC1 and FaSdhC2 in mycelial growth, sporulation, virulence, DON biosynthesis and sensitivity to SDHI fungicides in *F. asiaticum*. Additionally, we confirmed that six genetic variations in the SdhB, SdhC1 and SdhD subunits confer resistance to SDHI fungicides in *F. asiaticum*. Based on these findings, we propose an innovative strategy: developing FaSdhC2 as a novel drug target. In contrast to conventional broad‐spectrum SDHI fungicides, the design of specific inhibitors that selectively target the FaSdhC2 subunit could allow for more precise targeting against *F. asiaticum*. Using these specific inhibitors along with existing SDHI fungicides could enhance the efficacy of the SDHI fungicides and mitigate the risk of resistance development. This strategy could lead to a sustainable and effective way to manage plant diseases caused by *Fusarium* species.

## 2 Results

### 2.1 Identification and Knockout of FaSdhC1 and FaSdhC2 in *F. asiaticum*

**FIGURE 1:**
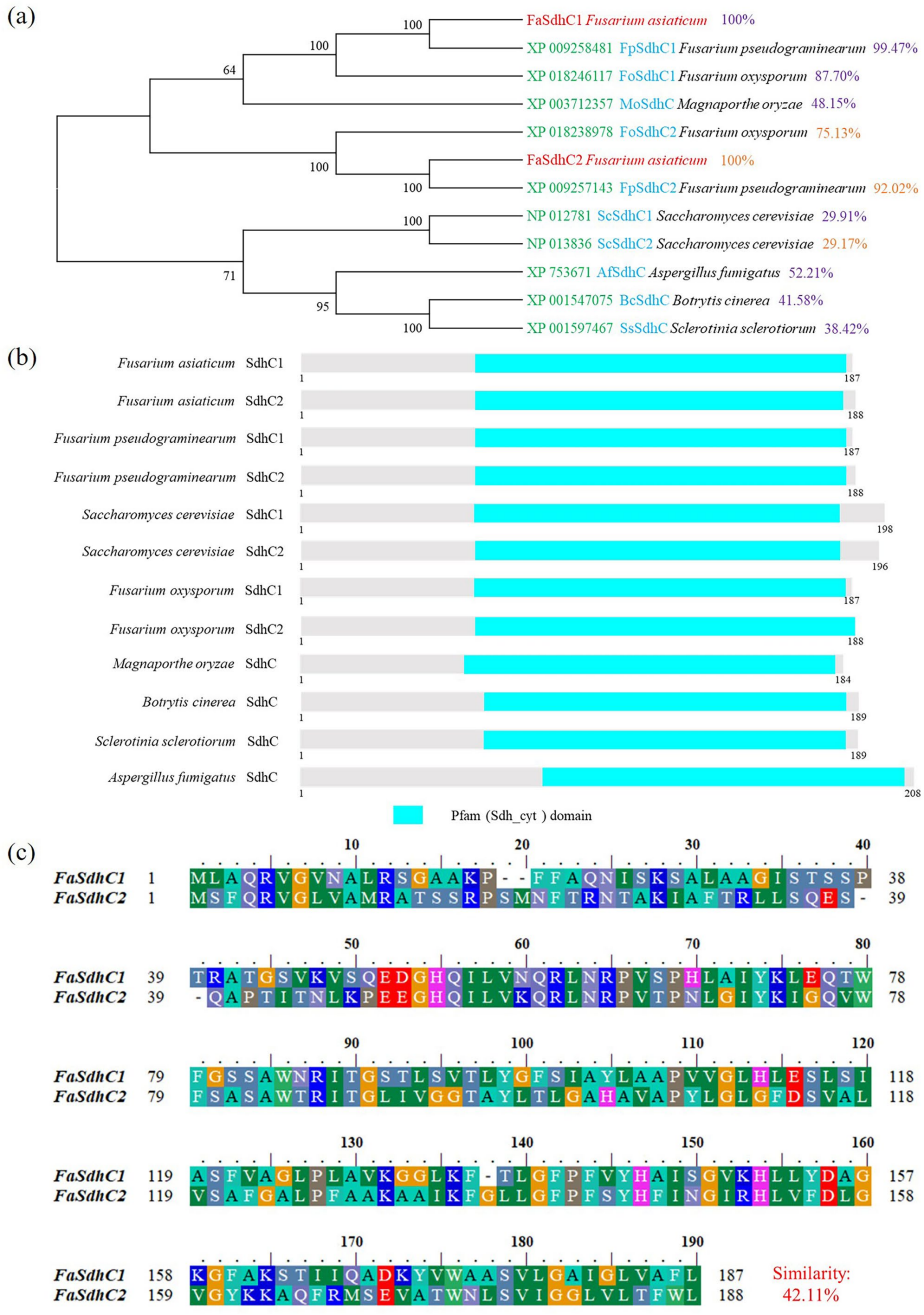
Identification and analysis of FaSdhC1 and FaSdhC2 in *Fusarium asiaticum*. (a) Phylogenetic trees of the SdhC subunit homologues in *F. asiaticum* and other fungi. Using the neighbour‐joining method, a phylogenetic tree was constructed based on the amino acid sequences of SdhC subunits from eight common plant‐pathogenic fungi using MEGA 7.0 software. (b) Functional domain analysis of SdhC subunits. The domains of the SdhC subunit from eight plant‐pathogenic fungi, including *F. asiaticum*, were identified using the InterPro website (https://www.ebi.ac.uk/interpro/). All the SdhC subunits contained a Pfam domain. (c) Amino acid sequence alignment of FaSdhC1 and FaSdhC2 in *F. asiaticum*. The BLAST analysis of amino acid sequences was primarily conducted using BioEdit software.

Succinate dehydrogenase (SDH) is a crucial enzyme complex in the mitochondrial respiratory electron transport chain and plays an essential role in fungal cellular metabolism. To gain further insights into the SDH complex in *F. asiaticum*, we conducted BLAST analysis to identify its SDH subunits by comparing them with those of several well‐characterized filamentous plant‐pathogenic fungi, including *Saccharomyces cerevisiae* , *Magnaporthe oryzae*, *Fusarium pseudograminearum*, *F. graminearum*, *Fusarium oxysporum*, *Botrytis cinerea*, *Sclerotinia sclerotiorum* and *Aspergillus fumigatus* (Table S1). Our analysis revealed functional differentiation within the SdhC subunit of *F. asiaticum*, which consists of two homologous subunits, FaSdhC1 and FaSdhC2. The subunit exhibiting higher sequence homology to SdhC proteins in other pathogenic fungi was designated FaSdhC1, whereas the subunit with lower homology was named FaSdhC2 (Li et al. 2025). Sequence characterization indicated that the *FaSDHC1* gene spans 675 base pairs, contains two introns and encodes a protein of 187 amino acids. In comparison, *FaSDHC2* is 664 base pairs in length, also includes two introns, and encodes a protein of 188 amino acids. The overall sequence similarity between FaSdhC1 and FaSdhC2 proteins was found to be only 42.11% (Figure 1c).

To conduct a more comprehensive analysis of the SdhC subunits in *F. asiaticum*, a phylogenetic tree (Figure 1a) was constructed to elucidate their evolutionary relationships with homologous subunits from other filamentous fungi. The results indicated that the SdhC subunits of *F. asiaticum* exhibit low sequence similarity to those of *S. cerevisiae* , with identity values of 29.91% for SdhC1 and 29.17% for SdhC2 (Figure 1a). In contrast, high sequence conservation was observed among SdhC subunits within the *Fusarium* genus, with similarity exceeding 85% across species (Figure 1a). This differentiation pattern suggests a distinct evolutionary trajectory specific to the *Fusarium* lineage. Furthermore, protein domain analysis (http://smart.embl‐heidelberg.de/) of SdhC subunits from various pathogenic fungi revealed that both FaSdhC1 and FaSdhC2 harbour a conserved Pfam (Sdh_cyt) domain, which is crucial for the catalytic function of succinate dehydrogenase/fumarate reductase. This functional domain is widely preserved across fungal species, as illustrated in Figure 1b.

**FIGURE 2:**
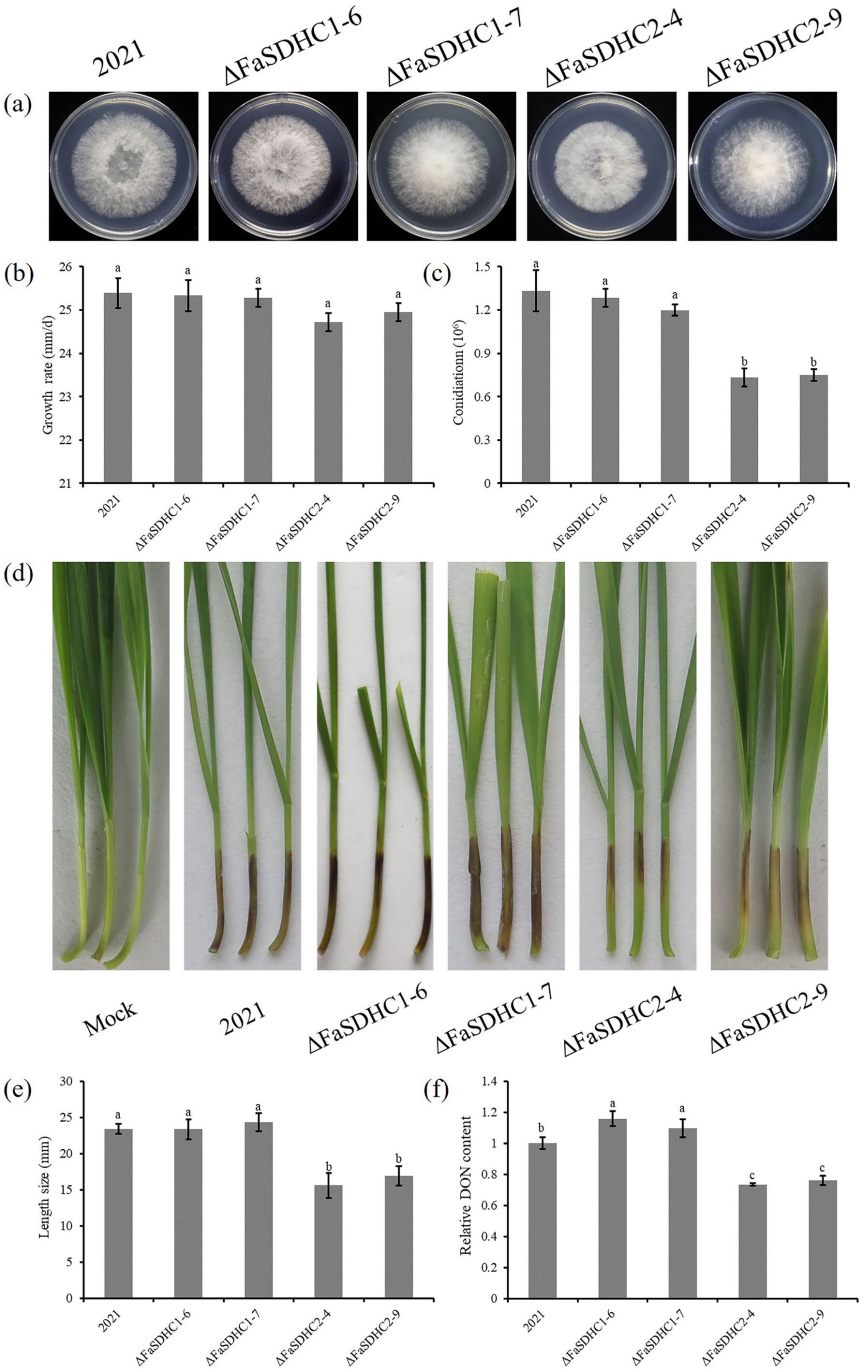
The regulatory role of the SdhC subunit in *Fusarium asiaticum* with respect to mycelial growth, sporulation, virulence and deoxynivalenol (DON) biosynthesis. (a) The growth phenotypes of *FaSDHC1* and *FaSDHC2* knockout mutants in *F. asiaticum*. (b) The growth rate of the *FaSDHC1* and *FaSDHC2* knockout mutants. All the strains were cultured on potato dextrose agar (PDA) for 3 days, and then their growth rates were measured. (c) Analysis of sporulation in *FaSDHC1* and *FaSDHC2* gene knockout mutants. After all the strains were cultivated in boiled mung bean (MBB) medium for 5 days, the spore production of each strain was measured. (d, e) The virulence of the *FaSDHC1* and *FaSDHC2* gene knockout mutants. The conidial concentrations of all strains were adjusted to 1 × 10^6^/mL. After inoculating 2 μL of the conidial suspension onto the wheat coleoptile, the infection lengths of the wheat coleoptile were measured within 10 days in a 25°C constant temperature incubator. (f) Analysis of DON production in *FaSDHC1* and *FaSDHC2* gene knockout mutants. After all the strains were cultured in the trichothecene biosynthesis induction (TBI) medium for 7 days, the filtrate was collected and the DON content was measured. The means of values represented by different lowercase letters are significantly different according to Fisher’s least significant difference test (*p* < 0.05).

To investigate the functions of *FaSDHC1* and *FaSDHC2*, a homologous recombination strategy was employed to generate targeted gene deletion mutants in *F. asiaticum*. For each gene, two independent deletion mutants were successfully obtained, and these mutants exhibited similar phenotypic profiles compared to wild‐type strain 2021 (Figure 2a). To confirm that the observed phenotypic changes in the ΔFaSDHC1 and ΔFaSDHC2 mutants were directly linked to the respective gene disruption, all mutants were validated using Southern blot analysis (Figure S1). Repeated attempts to generate a double mutant, ΔFaSDHC1ΔFaSDHC2, were unsuccessful, indicating that simultaneous deletion of both genes may result in lethality.

### 2.2 FaSdhC2 Is Involved in Regulating Sporulation, Virulence and DON Biosynthesis

To clarify the regulatory roles of FaSdhC1 and FaSdhC2 in *F. asiaticum*, we conducted a systematic investigation into vegetative growth, sporulation, virulence and DON biosynthesis in targeted deletion mutants. For vegetative growth, the *FaSDHC1* deletion mutants ΔFaSDHC1‐6, ΔFaSDHC1‐7 and *FaSDHC2* deletion mutants ΔFaSDHC2‐4 and ΔFaSDHC2‐9 exhibited no significant difference in growth phenotype and mycelial growth rate compared to the wild‐type strain 2021 (Figure 2a,b), indicating that deleting either FaSdhC1 or FaSdhC2 alone does not affect mycelial growth. For sporulation capacity, the *FaSDHC2* deletion mutants ΔFaSDHC2‐4 and ΔFaSDHC2‐9 produced significantly fewer conidia than the wild‐type strain 2021 (Figure 2c), whereas the *FaSDHC1* deletion mutants ΔFaSDHC1‐6 and ΔFaSDHC1‐7 showed no difference. These findings indicate that FaSdhC2 specifically regulates sporulation capability in *F. asiaticum*. In wheat coleoptile infection assays, the *FaSDHC2* deletion mutants ΔFaSDHC2‐4 and ΔFaSDHC2‐9 showed significantly reduced virulence 10 days after inoculation with conidial suspensions, whereas the *FaSDHC1* deletion mutants ΔFaSDHC1‐6 and ΔFaSDHC1‐7 exhibited similar virulence to the wild‐type strain 2021. This suggests that FaSdhC2 plays a crucial role in plant infection, whereas FaSdhC1 appears to be less important for virulence (Figure 2d,e). Additionally, considering that DON serves as a critical virulence factor, DON production was quantified using an ELISA. The ΔFaSDHC2 mutants showed a significant reduction in DON production, whereas the ΔFaSDHC1 mutants exhibited elevated DON content compared to the wild‐type strain 2021. These findings demonstrate that FaSdhC2 positively regulates DON biosynthesis, and the impaired virulence observed in the ΔFaSDHC2 mutants is correlated with their reduced DON production (Figure 2f). In summary, FaSdhC2 plays a vital role in regulating sporulation, virulence and DON biosynthesis in *F. asiaticum*, whereas FaSdhC1 appears to primarily modulate DON biosynthesis without affecting other pathogenic traits.

### 2.3 FaSdhC1 and FaSdhC2 Regulate Sensitivity to SDHI Fungicides

To elucidate the roles of FaSdhC1 and FaSdhC2 in modulating sensitivity to SDHI fungicides, we examined the sensitivity of the mutants ΔFaSDHC1‐6, ΔFaSDHC1‐7, ΔFaSDHC2‐4 and ΔFaSDHC2‐9 to three SDHI fungicides: boscalid, fluopyram and pydiflumetofen. The results revealed distinct phenotypic variations: the ΔFaSDHC1 mutants (ΔFaSDHC1‐6 and ΔFaSDHC1‐7) displayed significantly diminished sensitivity to three SDHI fungicides in comparison to the wild‐type strain 2021. Conversely, the ΔFaSDHC2 mutants (ΔFaSDHC2‐4 and ΔFaSDHC2‐9) exhibited a markedly increased sensitivity to these fungicides (Table 1). These findings indicate that FaSdhC1 and FaSdhC2 may have regulatory effects on the sensitivity to SDHI fungicides. Specifically, FaSdhC1 appears to function in a negative regulatory capacity, whereas FaSdhC2 demonstrates a positive regulatory influence. This antagonistic function relationship highlights a complex functional divergence between these paralogous subunits in the SDH complex in fungicide response pathways.

**TABLE 1:** The sensitivity of ΔFaSDHC1 and ΔFaSDHC2 to boscalid, fluopyram and pydiflumetofen.

Strains	EC_50_ (μg/mL)		
	Boscalid	Fluopyram	Pydiflumetofen
2021 (wild type)	35.05 ± 2.13 b	2.27 ± 0.49 b	0.0393 ± 0.0018 b
ΔFaSDHC1‐6	> 160 a	9.11 ± 0.61 a	0.0739 ± 0.0029 a
ΔFaSDHC1‐7	> 160 a	8.94 ± 0.43 a	0.0676 ± 0.0073 a
ΔFaSDHC2‐4	0.3350 ± 0.0357 c	0.0218 ± 0.0065 c	0.0046 ± 0.0002 c
ΔFaSDHC2‐9	0.2867 ± 0.0192 c	0.0133 ± 0.0014 c	0.0052 ± 0.0003 c

*Note:* All data were analysed statistically using the mean ± SE. The Fisher's LSD test (*p* = 0.05) was employed to assess significant differences in EC_50_ values among the groups. Groups that share the same letter indicate no significant difference between them.

### 2.4 Generation and Resistance Stability of Boscalid‐Resistant Mutants Derived From the ΔFaSDHC2 Mutant

To lower the pressure threshold for screening and facilitate the acquisition of a broader range of resistance mutations for exploring the resistance mechanisms of *F. asiaticum* to SDHI fungicides, we used the *FaSDHC2* gene knockout mutant ΔFaSDHC2‐9 as the parental strain and conducted fungicide taming and screening for boscalid‐resistant mutants, ultimately obtaining 11 stable boscalid‐resistant mutants: ΔFaSDHC2‐R24, ΔFaSDHC2‐R40, ΔFaSDHC2‐R41, ΔFaSDHC2‐R42, ΔFaSDHC2‐R58, ΔFaSDHC2‐R67, ΔFaSDHC2‐R81, ΔFaSDHC2‐R84, ΔFaSDHC2‐R112, ΔFaSDHC2‐R127 and ΔFaSDHC2‐R128. During the resistance induction process, 3000 mycelial plugs were inoculated onto the medium supplemented with boscalid, resulting in the successful acquisition of these 11 mutants. The frequency of resistance mutations was calculated to be 3.67 × 10^−3^. Each resistant mutant was serially subcultured 10 times on YBA plates, and their EC_50_ and resistance factor (RF) values were measured to evaluate fungicide sensitivity. Following 10 consecutive transfers, the RF values of these mutants remained largely unchanged, indicating a stable resistance phenotype to boscalid (Table 2). Notably, among these mutants, only ΔFaSDHC2‐R58 exhibited an RF value exceeding 100, whereas all other mutants displayed RF values > 200 (Table 2).

**TABLE 2:** The resistance stability of boscalid‐resistant strains from ΔFaSDHC2 after 1st and 10th serial passages on YBA plates supplemented with boscalid.

Strain	Phenotype^a^	EC_50_ (μg/mL)		RF^b^	
		1st	10th	1st	10th
ΔFaSDHC2‐9	S	0.3021 ± 0.0204 e	0.2851 ± 0.0165 e	—	—
ΔFaSDHC2‐R24	R	69.82 ± 2.61 b	65.57 ± 1.88 b	231.11	229.99
ΔFaSDHC2‐R40	R	72.89 ± 3.63 b	67.83 ± 2.91 b	241.28	237.92
ΔFaSDHC2‐R41	R	> 80 a	> 80 a	> 264.81	> 280.60
ΔFaSDHC2‐R42	R	68.48 ± 4.26 b	66.65 ± 3.12 b	226.80	233.78
ΔFaSDHC2‐R58	R	33.27 ± 2.26 d	30.71 ± 2.42 d	110.13	107.72
ΔFaSDHC2‐R67	R	> 80 a	> 80 a	> 264.81	> 280.60
ΔFaSDHC2‐R81	R	67.33 ± 5.47 bc	65.27 ± 3.95 b	222.87	228.94
ΔFaSDHC2‐R84	R	69.07 ± 3.29 b	65.36 ± 1.98 b	228.63	229.25
ΔFaSDHC2‐R112	R	66.53 ± 3.15 bc	64.69 ± 2.16 b	220.23	226.90
ΔFaSDHC2‐R127	R	61.76 ± 3.14 c	59.52 ± 3.34 c	204.44	208.77
ΔFaSDHC2‐R128	R	68.00 ± 5.41 bc	62.82 ± 2.95 bc	225.09	220.34

^a^ R represents resistance to boscalid, and S represents sensitivity to boscalid.

^b^ Resistance factor (RF): EC_50_ ratio of resistant mutants to their respective parental strains.

^c^ Means in a column followed by the same letter were not different according to Fisher's least significant difference (LSD) test (*p* = 0.05).

### 2.5 Identification and Analysis of Resistant Mutated Genotypes

**FIGURE 3:**
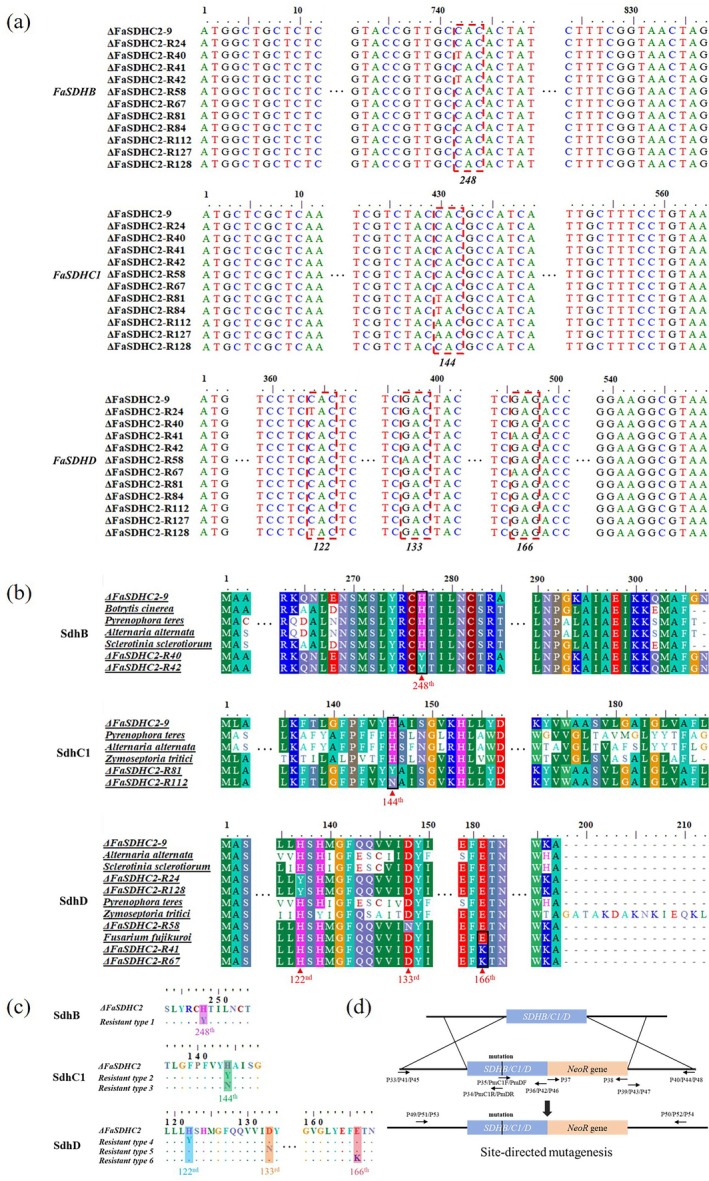
Identification and analysis of the mutant genotypes of the boscalid‐resistant strains derived from ΔFaSdhC2‐9. (a) Analysis of the coding sequences of SdhB, SdhC1 and SdhD in the boscalid‐resistant strains derived from ΔFaSdhC2‐9. The sequencing work was carried out by Shenggong Biotechnology Co. Ltd., and the sequence alignment analysis was accomplished using the BioEdit software. (b) The analysis comparing amino acid sequences of the boscalid‐resistant strain derived from ΔFaSdhC2‐9 with pathogenic fungi exhibiting the same mutation site. The amino acid sequence of the SDH subunit of *Fusarium asiaticum* was obtained through sequencing analysis. The amino acid sequences of the SDH subunits of other plant pathogens were obtained from the NCBI database. The amino acid sequence alignment was completed using the BioEdit software. (c) The amino acid mutant genotype of the boscalid‐resistant strains derived from ΔFaSdhC2‐9. (d) Construction of site‐directed mutagenesis vectors. The list of primers related to vector construction is presented in Table S4.

To elucidate the resistance mechanisms to SDHI fungicides, we cloned and sequenced the target genes encoding the subunits of succinate dehydrogenase complex, specifically the SdhB, SdhC1 and SdhD, from the 11 resistant mutants. Sequence analysis revealed multiple point mutations associated with resistance. In the FaSdhB subunit, a C‐to‐T transition (CAC → TAC) at position 742 was identified in the mutants ΔFaSDHC2‐R40 and ΔFaSDHC2‐R42, resulting in an amino acid substitution at codon 248 from histidine to tyrosine (H248Y) (Figure 3a). In the FaSdhC1 subunit, either a C‐to‐T or C‐to‐A substitution (CAC → TAC/AAC) at position 430 was observed in the mutants ΔFaSDHC2‐R81, ΔFaSDHC2‐R84, ΔFaSDHC2‐R112 and ΔFaSDHC2‐R127, leading to the replacement of histidine at codon 144 with tyrosine (H144Y) or asparagine (H144N) (Figure 3a). In the FaSdhD subunit, a C‐to‐T mutation (CAC → TAC) at position 364 was detected in the mutants ΔFaSDHC2‐R24 and ΔFaSDHC2‐R128, causing a substitution of histidine to tyrosine at codon 122 (H122Y) (Figure 3a). Additionally, a G‐to‐A mutation (GAC → AAC) at position 397 was detected in the mutant ΔFaSDHC2‐R58, resulting in the substitution of aspartic acid with asparagine at codon 133 (D133N) (Figure 3a). Furthermore, a G‐to‐A mutation (GAG → AAG) at position 496 was found in the mutants ΔFaSDHC2‐R41 and ΔFaSDHC2‐R67, leading to the replacement of glutamic acid with lysine at codon 166 (E166K) (Figure 3a). Collectively, these findings identify six distinct resistance‐associated genotypes: FaSdhB‐H248Y, FaSdhC1‐H144N, FaSdhC1‐H144N, FaSdhD‐H122Y, FaSdhD‐D133N and FaSdhD‐E166K (Figure 3c).

### 2.6 Biological Fitness of Boscalid‐Resistant Mutants Derived From ΔFaSDHC2

**FIGURE 4:**
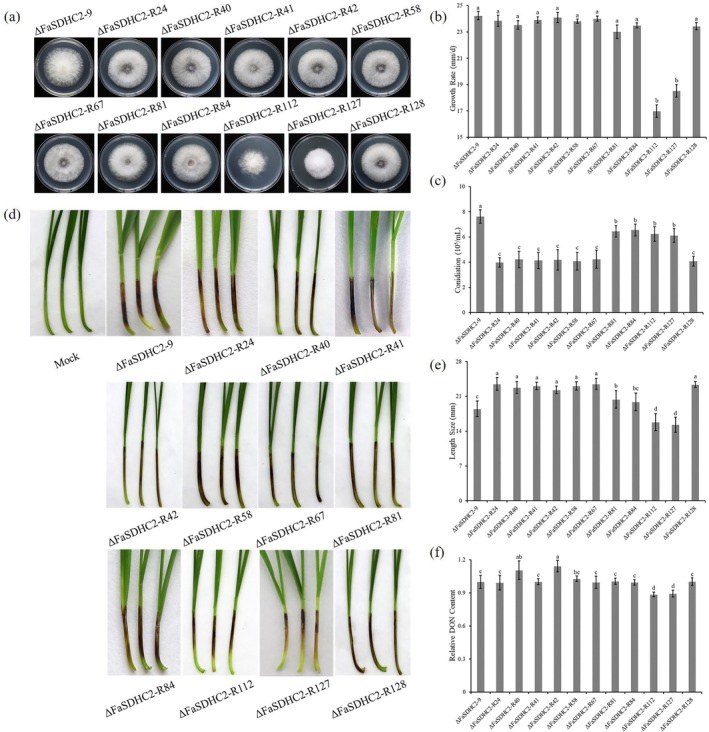
The biological fitness of the boscalid‐resistant strains derived from ΔFaSDHC2‐9. (a, b) The growth phenotype and growth rate of the boscalid‐resistant strains derived from ΔFaSDHC2‐9. All the strains were cultured on potato dextrose agar (PDA) for 3 days, and then their growth rates were measured. (c) The spore production of the boscalid‐resistant strains derived from ΔFaSDHC2‐9. After all the strains were cultivated in boiled mung bean (MBB) medium for 5 days, the spore production of each strain was measured. (d, e) The virulence of the boscalid‐resistant strains derived from ΔFaSDHC2‐9. The conidial concentrations of all strains were adjusted to 1 × 10^6^/mL. After inoculating 2 μL of the conidial suspension onto the wheat coleoptile, the infection lengths of the wheat coleoptile were measured within 10 days in a 25°C constant temperature incubator. (f) Analysis of deoxynivalenol (DON) production in boscalid‐resistant strains derived from ΔFaSDHC2‐9. After all the strains were cultured in trichothecene biosynthesis induction (TBI) medium for 7 days, the filtrate was collected and the DON content was measured. The means of values represented by different lowercase letters are significantly different according to Fisher’s least significant difference test (*p* < 0.05).

To gain a more comprehensive understanding of the biological fitness of boscalid‐resistant mutants derived from ΔFaSDHC2, we evaluated key physiological and pathogenic traits, including mycelial growth, sporulation capability, virulence and DON biosynthesis, across multiple boscalid‐resistant mutants. A 3‐day cultivation assay was conducted under controlled conditions at a constant temperature of 25°C to assess their mycelial growth in all mutants relative to the parental strain ΔFaSDHC2‐9. The results indicated that only the ΔFaSDHC2‐R112 and ΔFaSDHC2‐R127 mutants exhibited a reduction in mycelial growth, whereas other resistant mutants displayed mycelial growth levels comparable to those of the parental strain (Figure 4a,b). To further investigate the sporulation capability and virulence of the boscalid‐resistant mutants, we quantified sporulation in boiled mung bean (MBB) medium and evaluated lesion development on wheat coleoptiles. The results show that, compared to the parental strain ΔFaSDHC2‐9, all mutants exhibited significantly decreased sporulation capability (Figure 4c), indicating that the sporulation capacity of the ΔFaSDHC2‐9 strain was impaired during the evolution of resistance. In wheat coleoptile infection assays, the virulence of the mutants ΔFaSDHC2‐R112 and ΔFaSDHC2‐R117 was found to be significantly lower than that of the parental strain ΔFaSDHC2‐9. Conversely, the virulence of the remaining mutants showed a noticeable increase relative to the parental strain ΔFaSDHC2‐9, but there was no significant difference compared to the wild‐type strain 2021 (Figures 2d,e and 4d,e). Additionally, DON production in the resistant mutants was evaluated using a DON detection kit. The results showed that only the mutants ΔFaSDHC2‐R40 and ΔFaSDHC2‐R42 exhibited a significant increase in DON content in comparison to the parental strain ΔFaSDHC2‐9, and there was no significant difference compared to the wild‐type strain 2021 (Figures 2f and 4f). In contrast, the mutants ΔFaSDHC2‐R112 and ΔFaSDHC2‐R127 showed a notable reduction in DON content compared to the parental strain ΔFaSDHC2‐9. Furthermore, the remaining mutants did not show any significant difference in DON content when compared to ΔFaSDHC2‐9 (Figure 4f).

The results of the biological fitness assessment indicate that all resistant mutants displayed varying degrees of biological fitness changes, predominantly characterized by a significant decline in sporulation capability across the resistant mutants. This reduction may reflect a fitness penalty associated with the acquisition of resistance. Notably, the mutants ΔFaSDHC2‐R112 and ΔFaSDHC2‐R127 showed a notable decline in biological fitness, as evidenced by decreased mycelial growth, impaired sporulation capability and attenuated DON biosynthesis. Genotypic analyses suggest that this phenotypic decline is potentially attributable to the FaSdhC1‐H144N mutation, which appears to negatively affect mycelial growth, sporulation and DON biosynthesis.

### 2.7 Cross‐Resistance Patterns Among Resistant Genotypes

To enhance our understanding of the responses of boscalid‐resistant mutants to different fungicides, we evaluated the sensitivity to selected SDHI fungicides, boscalid, fluopyram, pydiflumetofen, isopyrazam, benzovindiflupyr and the QoI fungicide pyraclostrobin. The results showed that the boscalid‐resistant mutants ΔFaSDHC2‐R40 and ΔFaSDHC2‐R42 exhibited resistance to pydiflumetofen, isopyrazam and benzovindiflupyr, but remained sensitive to fluopyram (Table 3). This differential sensitivity pattern is likely attributable to the specific mutation genotypes of these mutants, particularly the FaSdhB‐H248Y mutation, which has been confirmed to have high specificity for the sensitivity to fluopyram (Angelini et al. 2014; Sun et al. 2024). In contrast, other boscalid‐resistant mutants harbouring distinct mutation genotypes, such as FaSdhC1‐H144Y/N, FaSdhD‐H122Y, FaSdhD‐D133N and FaSdhD‐E166K, exhibited resistance to fluopyram, pydiflumetofen, isopyrazam and benzovindiflupyr. Notably, all tested resistant mutants remained sensitive to QoI fungicide pyraclostrobin. These findings indicate that, in *F. asiaticum*, boscalid‐resistant mutants carrying the FaSdhC1‐H144Y/N, FaSdhD‐H122Y, FaSdhD‐D133N or FaSdhD‐E166K genotypes exhibit resistance to multiple SDHI fungicides, including fluopyram, pydiflumetofen, isopyrazam and benzovindiflupyr, while remaining sensitive to QoI fungicides such as pyraclostrobin. In contrast, boscalid‐resistant mutants carrying FaSdhB‐H248Y genotype exhibit a distinct resistance profile: they are resistant to pydiflumetofen, isopyrazam and benzovindiflupyr, but remain sensitive to both SDHI fungicide fluopyram and QoI fungicide pyraclostrobin.

**TABLE 3:** The sensitivity of the boscalid‐resistant strains from ΔFaSDHC2 to boscalid and five other fungicides.

Strain	Phenotype	Genotype	EC_50_ (μg/mL)					
			Boscalid	Fluopyram	Pydiflumetofen	Isopyrazam	Benzovindiflupyr	Pyraclostrobin
ΔFaSDHC2‐9	S	—	0.2943 ± 0.0134 e	0.0254 ± 0.0126 b	0.0046 ± 0.0012 b	0.0054 ± 0.0021 c	0.0109 ± 0.0024 b	0.0563 ± 0.0103 ab
ΔFaSDHC2‐R24	R	SdhD‐H122Y	66.39 ± 2.91 bc	0.3918 ± 0.1158 a	0.0178 ± 0.0052 a	0.0352 ± 0.0093 ab	0.0672 ± 0.0093 a	0.0313 ± 0.0132 b
ΔFaSDHC2‐R40	R	SdhB‐H248Y	70.83 ± 4.87 b	0.0238 ± 0.0091 b	0.0175 ± 0.0052 a	0.0443 ± 0.0131 a	0.0432 ± 0.0099 a	0.0582 ± 0.0083 ab
ΔFaSDHC2‐R41	R	SdhD‐E166K	> 80 a	0.3362 ± 0.0928 a	0.0211 ± 0.0097 a	0.0472 ± 0.0156 a	0.0551 ± 0.0242 a	0.0593 ± 0.0097 ab
ΔFaSDHC2‐R42	R	SdhB‐H248Y	71.68 ± 3.11 b	0.0277 ± 0.0128 b	0.0192 ± 0.0093 a	0.0554 ± 0.0153 a	0.0467 ± 0.0072 a	0.0552 ± 0.0046 ab
ΔFaSDHC2‐R58	R	SdhD‐D133N	32.94 ± 3.05 d	0.2784 ± 0.0875 a	0.0229 ± 0.0089 a	0.0440 ± 0.0151 a	0.0512 ± 0.0112 a	0.0491 ± 0.0141 ab
ΔFaSDHC2‐R67	R	SdhD‐E166K	> 80 a	0.2853 ± 0.1168 a	0.0222 ± 0.0093 a	0.0395 ± 0.0109 ab	0.0538 ± 0.0133 a	0.0530 ± 0.0046 ab
ΔFaSDHC2‐R81	R	SdhC1‐H144Y	66.91 ± 3.77 bc	0.2795 ± 0.1012a	0.0191 ± 0.0075 a	0.0368 ± 0.0179 ab	0.0601 ± 0.0116 a	0.0336 ± 0.0087 b
ΔFaSDHC2‐R84	R	SdhC1‐H144Y	69.19 ± 4.60 bc	0.2676 ± 0.1003 a	0.0209 ± 0.0111 a	0.0310 ± 0.0137 ab	0.0584 ± 0.0147 a	0.0395 ± 0.0085 ab
ΔFaSDHC2‐R112	R	SdhC1‐H144N	64.55 ± 4.30 bc	0.2455 ± 0.0826 a	0.0233 ± 0.0124 a	0.0285 ± 0.0109 ab	0.0538 ± 0.0157 a	0.0656 ± 0.0185 a
ΔFaSDHC2‐R127	R	SdhC1‐H144N	61.40 ± 3.08 c	0.2696 ± 0.0845 a	0.0202 ± 0.0107 a	0.0237 ± 0.0110 ab	0.0493 ± 0.0144 a	0.0593 ± 0.0087 ab
ΔFaSDHC2‐R128	R	SdhD‐H122Y	68.68 ± 4.35 bc	0.3855 ± 0.1237 a	0.0218 ± 0.0118 a	0.0464 ± 0.0159 a	0.0615 ± 0.0077 a	0.0380 ± 0.0129 ab

^a^ Means in a column followed by the same letter were not different according to Fisher's least significant difference (LSD) test (*p* = 0.05).

### 2.8 Validation of Biological Fitness and Fungicide Sensitivity in Site‐Directed Mutants

**FIGURE 5:**
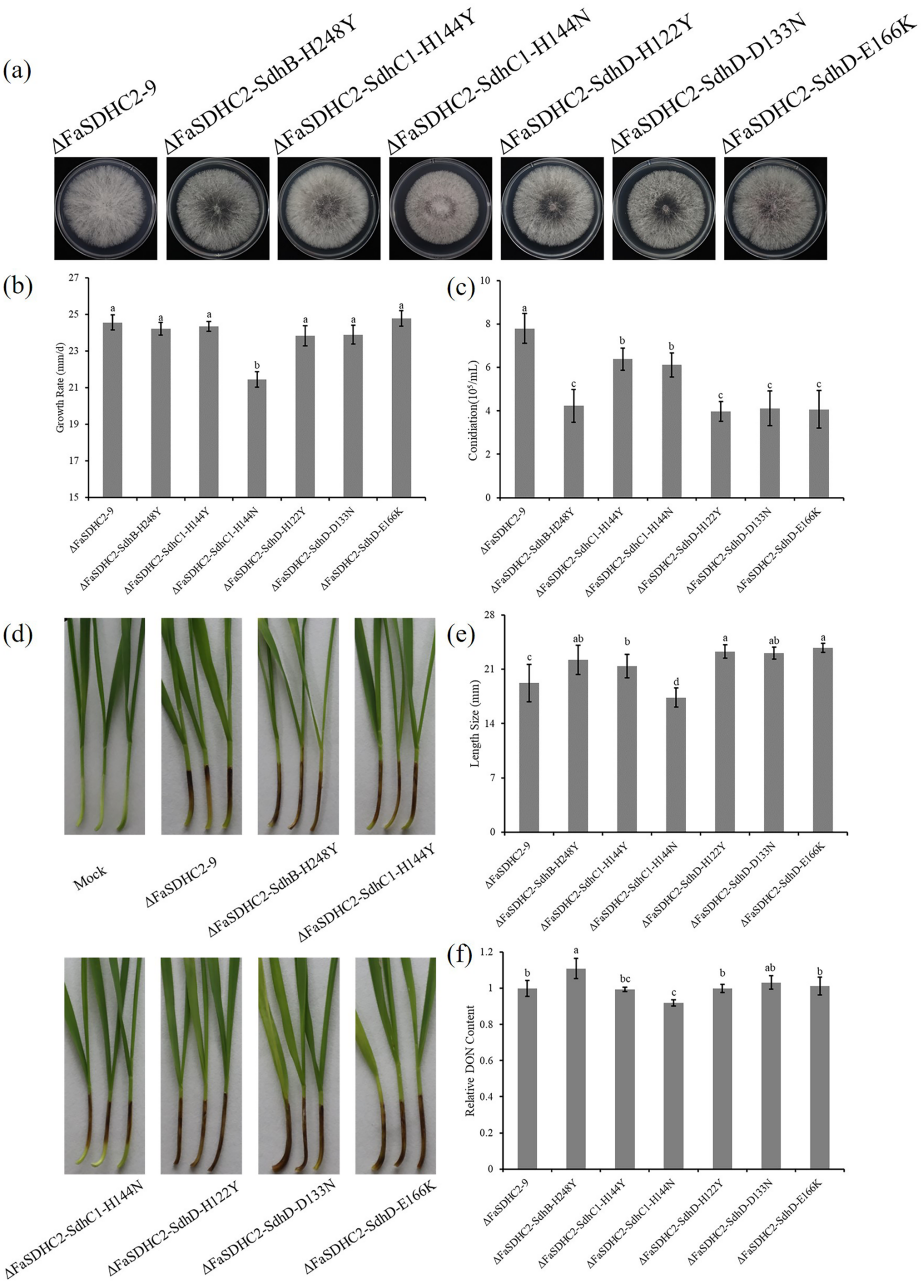
The biological fitness of the site‐directed mutants. (a, b) The growth phenotype and growth rate of the site‐directed mutants derived from ΔFaSDHC2‐9. All the strains were cultured on potato dextrose agar (PDA) for 3 days, and then their growth rates were measured. (c) The spore production of the site‐directed mutants derived from ΔFaSDHC2‐9. After all the strains were cultivated in boiled mung bean (MBB) medium for 5 days, the spore production of each strain was measured. (d, e) The virulence of the site‐directed mutants derived from ΔFaSDHC2‐9. The conidial concentrations of all strains were adjusted to 1 × 10^6^/mL. After inoculating 2 μL of the conidial suspension onto the wheat coleoptile, the infection lengths of the wheat coleoptile were measured within 10 days in a 25°C constant temperature incubator. (f) Analysis of deoxynivalenol (DON) production in site‐directed mutants derived from ΔFaSDHC2‐9. After all the strains were cultured in trichothecene biosynthesis induction (TBI) medium for 7 days, the filtrate was collected and the DON content was measured. The means of values represented by different lowercase letters are significantly different according to Fisher’s least significant difference test (*p* < 0.05).

To further demonstrate that these mutation genotypes are critical determinants of resistance to SDHI fungicides in the ΔFaSDHC2‐9 strain, we designed site‐directed mutagenesis vectors based on a homologous recombination strategy (Figure 3d). Through protoplast‐mediated transformation, we successfully obtained a series of site‐directed mutants. Following confirmation of the desired genetic modification by sequencing, one representative mutant for each mutation genotype was selected and designated as follows: ΔFaSDHC2‐SdhB‐H248Y, ΔFaSDHC2‐SdhC1‐H144Y, ΔFaSDHC2‐SdhC1‐H144N, ΔFaSDHC2‐SdhD‐H122Y, ΔFaSDHC2‐SdhD‐D133N and ΔFaSDHC2‐SdhD‐E166K. To comprehensively assess the biological fitness of the site‐directed mutants, we evaluated phenotypic traits, including mycelial growth, sporulation capability, virulence and DON biosynthesis. Mycelial growth analysis on PDA revealed that, in contrast to the parental strain ΔFaSDHC2‐9, only the ΔFaSDHC2‐SdhC1‐H144N mutant exhibited reduced mycelial growth (Figure 5a,b). In terms of sporulation capability, all site‐directed mutants displayed significantly lower sporulation capability compared to the parental strain (Figure 5c). Regarding virulence, a reduction was observed exclusively in the ΔFaSDHC2‐SdhC1‐H144N mutant, whereas other site‐directed mutants displayed enhanced virulence (Figure 5d,e). Additionally, the quantification of DON content via ELISA demonstrated a notable reduction in the ΔFaSDHC2‐SdhC1‐H144N mutant when compared to the parental strain. Conversely, there was an observed increase in DON content in the ΔFaSDHC2‐SdhB‐H248Y mutant relative to the parental strain. Importantly, no significant differences in DON content were detected among the other site‐directed mutants (Figure 5f). These findings suggest that the different mutation genotypes impair biological fitness to varying extents, with the FaSdhC1‐H144N mutation resulting in multifaceted fitness penalties.

We selected several SDHI fungicides, namely, boscalid, fluopyram, pydiflumetofen, isopyrazam and benzovindiflupyr, to evaluate the sensitivity of the site‐directed mutants towards these fungicides. The ΔFaSDHC2‐SdhB‐H248Y mutant showed high resistance to boscalid, low resistance to pydiflumetofen, isopyrazam and benzovindiflupyr, but remained sensitive to fluopyram. Conversely, the ΔFaSDHC2‐SdhC1‐H144Y, ΔFaSDHC2‐SdhC1‐H144N, ΔFaSDHC2‐SdhD‐H122Y, ΔFaSDHC2‐SdhD‐D133N and ΔFaSDHC2‐SdhD‐E166K mutants exhibited resistance to all tested SDHI fungicides, including boscalid, fluopyram, pydiflumetofen, isopyrazam and benzovindiflupyr (Table 4). These findings reveal the distinct cross‐resistance patterns among SDHI fungicides and suggest that the FaSdhB‐H248Y mutation does not confer resistance to fluopyram, highlighting its specificity in resistance development.

**TABLE 4:** The sensitivity of the site‐directed mutants from ΔFaSDHC2 to five succinate dehydrogenase inhibitor (SDHI) fungicides.

Strains	Phenotype	EC_50_ ^a^ (μg/mL)				
		Boscalid	Fluopyram	Pydiflumetofen	Isopyrazam	Benzovindiflupyr
ΔFaSDHC2‐9	S	0.3068 ± 0.0896 d	0.0150 ± 0.0062 b	0.0054 ± 0.0014 c	0.0046 ± 0.0018 d	0.0084 ± 0.0019 d
ΔFaSDHC2‐SdhB‐H248Y	R	71.72 ± 4.81 ab	0.0162 ± 0.0037 b	0.0171 ± 0.0040 b	0.0575 ± 0.0109 a	0.0435 ± 0.0051 c
ΔFaSDHC2‐SdhC1‐H144Y	R	68.04 ± 5.25 b	0.2953 ± 0.0917 a	0.0169 ± 0.0035 b	0.0287 ± 0.0104 bc	0.0604 ± 0.0098 ab
ΔFaSDHC2‐SdhC1‐H144N	R	62.63 ± 5.89 b	0.2872 ± 0.0997 a	0.0150 ± 0.0018 b	0.0229 ± 0.0086 c	0.0464 ± 0.0103 bc
ΔFaSDHC2‐SdhD‐H122Y	R	64.03 ± 5.54 b	0.4030 ± 0.0862 a	0.0175 ± 0.0030 b	0.0429 ± 0.0065 ab	0.0739 ± 0.0131 a
ΔFaSDHC2‐SdhD‐D133N	R	30.96 ± 5.40 c	0.2902 ± 0.0690 a	0.0255 ± 0.0065 a	0.0390 ± 0.0080 bc	0.0454 ± 0.0098 c
ΔFaSDHC2‐SdhD‐E166K	R	79.57 ± 6.08 a	0.2868 ± 0.0859 a	0.0198 ± 0.0019 a	0.0376 ± 0.0054 bc	0.0480 ± 0.0131 bc

^a^ Means in a column followed by the same letter were not different according to Fisher's least significant difference (LSD) test (*p* = 0.05).

### 2.9 Effects of FaSdhC1 or FaSdhC2 Deletions on SDH Enzyme Activity and the Binding Stability of SDHI Fungicides

**FIGURE 6:**
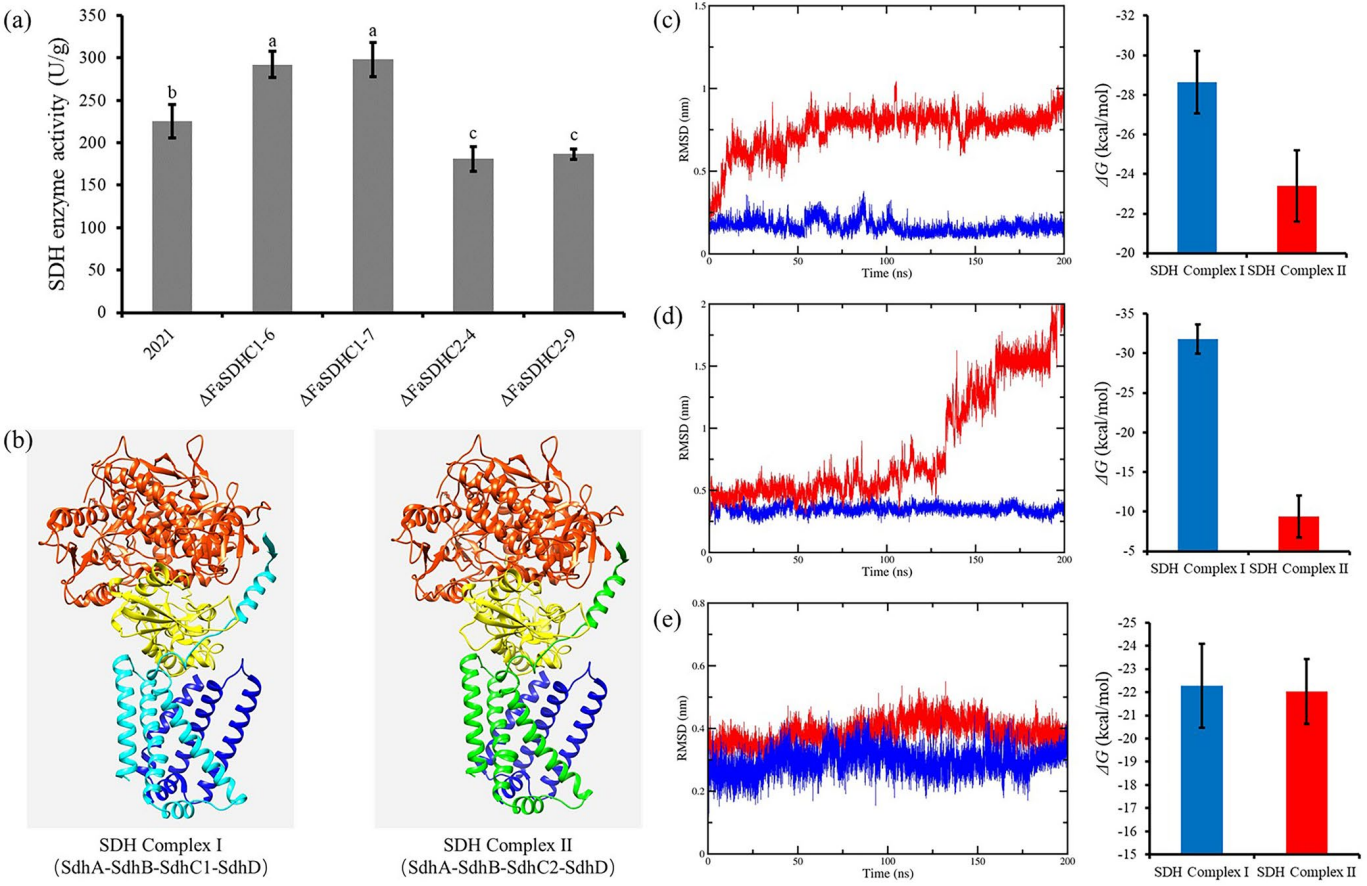
Succinate dehydrogenase (SDH) activity and molecular dynamics simulation. (a) Determination of SDH activity in *FaSDHC1* and *FaSDHC2* knockout mutants. After all the strains were cultured for 36 h in yeast extract‐peptone‐dextrose (YEPD) medium, their SDH activities were determined using the SDH activity detection kit. The means of values represented by different lowercase letters are significantly different according to Fisher’s least significant difference test (*p* < 0.05). (b) The SDH complex model in the *FaSDHC1* and *FaSDHC2* knockout mutants. The models of SDH complex I and SDH complex II were obtained through homology modelling on the AlphaFold website. The SDH complex model in the *FaSDHC1* and *FaSDHC2* gene knockout mutants. The models of SDH complex I and SDH complex II were obtained through homology modelling on the AlphaFold website. In the SDH complex, the orange colour represents the SdhA subunit, the yellow colour represents the SdhB subunit, the blue colour represents the SdhD subunit, and the cyan and green colours represent the SdhC1 and SdhC2 subunits respectively. (c–e) Molecular dynamics simulations of the binding of SDH complex I and SDH complex II to boscalid (c), fluopyram (d) and pydiflumetofen (e). In the results of root mean square deviation (RMSD) and the free binding energy Δ*G*, the blue and red colours represent the SDH complex I and the SDH complex II, respectively.

To investigate the differences in succinate dehydrogenase (SDH) activity between ΔFaSDHC1 and ΔFaSDHC2 mutants, we measured SDH activity using a specific assay kit. The results showed that deletion of *FaSDHC1* led to increased SDH activity, whereas deletion of *FaSDHC2* resulted in decreased SDH activity (Figure 6a).

To further elucidate the mechanistic basis for the differential sensitivity to SDHI fungicides in these two mutants, we performed molecular dynamics simulations to analyse the binding of three SDHI fungicides—boscalid, fluopyram and pydiflumetofen—to the SDH complex in the absence of FaSdhC1 and FaSdhC2, respectively. Using AlphaFold‐based homology modelling, we constructed three‐dimensional structures of the SDH complex under each SdhC subunit deletion background, designated as SDH Complex I (comprising FaSdhA, FaSdhB, FaSdhC1 and FaSdhD) and SDH Complex II (comprising FaSdhA, FaSdhB, FaSdhC2 and FaSdhD) (Figure 6b). Molecular dynamics simulations revealed that compared with SDH Complex II, SDH Complex I exhibited lower binding free energy and greater binding stability with boscalid and fluopyram, while no significant difference in binding stability with pydiflumetofen was observed between the two complexes (Figure 6c–e). Taken together with the SDH activity data, these findings suggest that deletion of *FaSDHC1* increases SDH activity but reduces the affinity of the SDH complex for SDHI fungicides, thereby decreasing fungicide sensitivity. Conversely, deletion of *FaSDHC2* reduces SDH activity while enhancing the affinity of the SDH complex for SDHI fungicides, leading to increased fungicide sensitivity.

## 3 Discussion

FHB poses a significant and widespread threat to global food production and security (Goswami and Kistler 2004). The absence of highly resilient wheat varieties has resulted in a considerable dependence on chemical control measures. However, the repeated use of the same type of fungicides has led to the gradual development of resistance (Duan et al. 2014, 2016; Zhao et al. 2022), making it urgent to develop new fungicides with novel mechanisms of action and chemical structures. SDHI fungicides have demonstrated significant efficacy in controlling various plant diseases in recent years. However, the extensive application of these fungicides has raised significant concerns regarding the emergence of resistance. This has prompted researchers to study the mechanisms underlying the development of resistance to SDHI fungicides in numerous pathogenic fungi (Wang et al. 2022; Song, Qiu, et al. 2024; Yin et al. 2024; Zhang et al. 2025; Li et al. 2025). It is known that the target of SDHI fungicides is the SDH complex, which consists of four core subunits: SdhA, SdhB, SdhC and SdhD. This study found that the SdhC subunit in *F. asiaticum* has genetic differentiation, forming two paralogous subunits: FaSdhC1 and FaSdhC2. By constructing single knockout mutants, we discovered that FaSdhC1 and FaSdhC2 have opposite regulatory effects on the sensitivity to SDHI fungicides: the absence of FaSdhC1 leads to a decrease in sensitivity, while the absence of FaSdhC2 leads to an increase in sensitivity. This finding reveals a new mechanism by which fungi finely regulate fungicides response through subunit functional differentiation.

**FIGURE 7:**
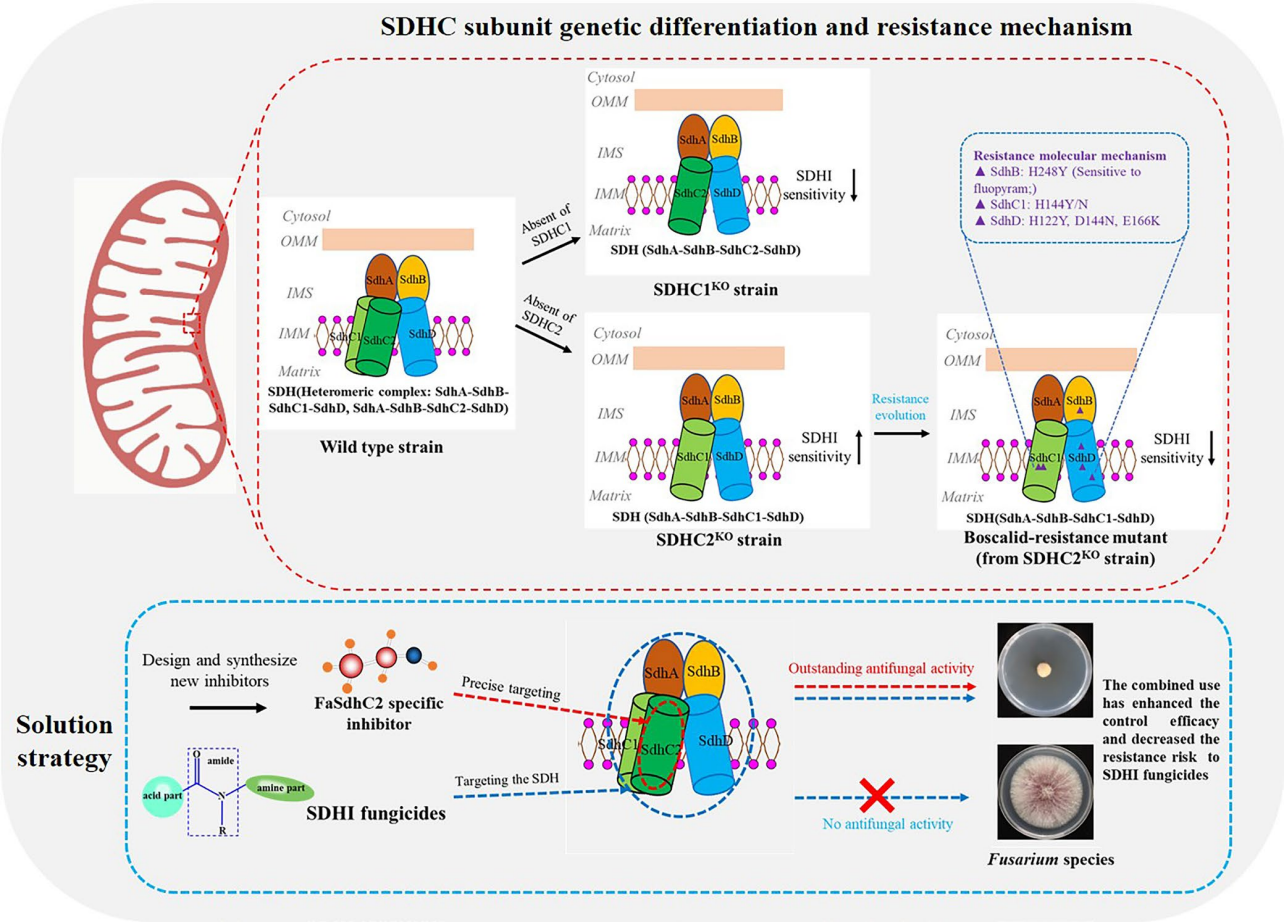
Model of the succinate dehydrogenase (SDH) complex in *Fusarium* species and new solution strategies. Based on our findings, we propose that there are two distinct heterologous SDH complex models in *Fusarium* species: SdhA‐SdhB‐SdhC1‐SdhD and SDH SdhA‐SdhB‐SdhC2‐SdhD. Notably, these complexes exhibit varying binding affinities for SDHI fungicides, resulting in differences in sensitivity. After the absence of SdhC2, the SdhA‐SdhB‐SdhC1‐SdhD was able to maintain the basic functions of the SDH enzyme, thereby establishing a resistance mechanism similar to that observed in the wild‐type strain. Furthermore, we propose developing FaSdhC2‐specific inhibitors as a targeted strategy against *Fusarium* species. When used in conjunction with SDHI fungicides, these inhibitors would have the potential to enhance the control efficacy against these pathogens, while concurrently mitigating the risk of developing resistance. In the solution strategy, the blue arrow shows that SDHI fungicides alone do not exhibit antifungal activity against *Fusarium* species. The red and blue double arrow shows that combining the FaSdhC2‐specific inhibitor with the SDHI fungicide produces outstanding antifungal activity against *Fusarium* species.

Previous studies have shown that the impact of SdhC subunit variations on the sensitivity to SDHI fungicides is highly species‐specific (Wang et al. 2022; Steinhauer et al. 2020; Shao et al. 2020). For example, the variation of SdhC in *B. cinerea* results in differing sensitivities among its populations to SDHI fungicides (Shao et al. 2020). In *Z. tritici*, there is an alt‐SdhC subtype that confers resistance to SHA class SDHI fungicides (Steinhauer et al. 2020); while in *S. sclerotiorum*, SdhC1 and SdhC2 did not show significant differences in SDHI fungicides sensitivity (Wang et al. 2022). This study further enriches this content: In *F. asiaticum*, FaSdhC1 and FaSdhC2 not only cannot be functionally substituted for each other but also exhibit opposite regulatory directions. Molecular dynamics simulations explain this phenomenon from a physical and chemical perspective: the complex containing FaSdhC1 has a significantly stronger binding stability with SDHI fungicides than the complex containing FaSdhC2, suggesting that FaSdhC2 may reduce the affinity of the complex for SDHI fungicides by changing the conformation of the drug binding pocket, thereby conferring higher basal resistance to the fungus. Based on these findings, we propose that developing specific inhibitors targeting FaSdhC2 and using them in combination with existing SDHI fungicides would be expected to achieve synergistic effects and delay the development of resistance by reducing the baseline resistance threshold of fungi and expanding the spectrum of resistance screening mutations (Figure 7). The advantages of this strategy include: (1) enhancing selective inhibition against the pathogen by targeting distinct subunits of complex II; (2) alleviating the selection pressure imposed by single SDHI fungicides, thereby ‘sensitizing’ existing agents and restoring their efficacy against certain resistant strains; and (3) compelling the pathogenic fungus to evolve through a more costly adaptive pathway, thus curbing the rapid expansion of resistant populations under field conditions. Nevertheless, the implementation of this strategy presents several challenges. First, small‐molecule inhibitors targeting FaSdhC2 must exhibit high specificity to minimize off‐target effects on non‐target organisms, such as soil microbiota, crops and mammals. Second, the risk of resistance evolution under combination therapy requires systematic evaluation, as prolonged use may trigger mutations in other complex II subunits or activate compensatory metabolic pathways. Furthermore, practical considerations—including the stability and efficacy of candidate inhibitors, the cost of field application and compatibility with existing fungicides—are the key obstacles in the process of transferring this approach from laboratory settings to field application. Future efforts should therefore prioritize the screening and structural optimization of highly specific inhibitors, alongside the integration of resistance monitoring and predictive risk models to assess the long‐term sustainability of this combination strategy.

An important corollary of the strategy is that the existence of FaSdhC2 has increased the basal tolerance threshold of the wild‐type strain to SDHI fungicides, thereby masking possible mutations on other subunits. To verify this corollary, we used the hypersensitive strain ΔFaSdhC2‐9 as the starting strain and screened for resistance mutants under a lower selection pressure, successfully obtaining 11 stably inherited resistance mutants. Target gene sequencing revealed six mutant genotypes: FaSdhB‐H248Y, FaSdhC1‐H144Y, FaSdhC1‐H144N, FaSdhD‐H122Y, FaSdhD‐D133N and FaSdhD‐E166K. Among them, FaSdhB‐H248Y and FaSdhD‐E166K have been reported in *F. asiaticum* and *F. fujikuroi* (Chen et al. 2021; Liu et al. 2023), while FaSdhC1‐H144Y/N, FaSdhD‐H122Y and FaSdhD‐D133N were reported for the first time in the *Fusarium* genus. By constructing site‐directed mutants and determining their sensitivity, we confirmed that these mutations were the direct cause of resistance in ΔFaSdhC2‐9. This result strongly supports our corollary: the deletion of FaSdhC2 is equivalent to removing a buffer barrier, allowing those mutations that could not occur under the selection pressure of the wild‐type background to be screened out. This indicates that the differentiation state of the SdhC subunit directly determines the width and direction of the fungal resistance evolution path.

Under the ΔFaSDHC2 genetic background, we identified four novel mutant genotypes: FaSdhC1‐H144Y, FaSdhC1‐H144N, FaSdhD‐H122Y and FaSdhD‐D133N. Sequence alignment revealed that these mutation sites (FaSdhC1‐H144, FaSdhD‐H122/D133) are situated within highly conserved functional regions of the SDH complex, and corresponding resistance mutations have been documented in other pathogenic fungi, including *Pyrenophora teres* , *Alternaria alternata* / *A. solani* , *S. sclerotiorum* and *Z. tritici*. For instance, FaSdhC1‐H144Y/N corresponds to SdhC‐H134R in *P. teres* , SdhC‐H134R/Q in *A. alternata/A. solani* and SdhC‐H152R in *Z. tritici* (Sautua and Carmona 2023; Metz et al. 2019; Dooley et al. 2016); FaSdhD‐H122Y corresponds to SdhD‐H134R in *P. teres* , SdhD‐H133R in *A. solani* and SdhD‐H132R in *S. sclerotiorum* (Putsepp et al. 2024; Mallik et al. 2014; Wang et al. 2015); and FaSdhD‐D133N corresponds to SdhD‐D145G in *P. teres* and SdhD‐D129G in *Z. tritici* (Sautua and Carmona 2023; FRAC, available at https://www.frac.info). These findings further support the critical role of these amino acid residues in SDHI binding. Notably, when the fungus relies exclusively on the FaSdhC1 complex (complex I), its resistance threshold to SDHI fungicides is lowered, thereby altering the mutational plasticity of other SDH subunits and enabling the selection of rare mutations that are typically not observed in the wild‐type background. Collectively, these results indicate that while the resistance evolution pathway is influenced by the FaSdhC2 subunit background, mutation hotspots remain concentrated within conserved functional domains.

The adaptive cost of resistance mutations is the core indicator for assessing the field resistance risk. This study systematically evaluated the mycelial growth, sporulation, virulence and DON biosynthesis of each mutant. The results showed that the FaSdhC1‐H144N mutation led to slowed mycelial growth, reduced sporulation, and decreased DON synthesis, while the FaSdhB‐H248Y mutation increased virulence and DON synthesis, but reduced sporulation. Other mutations (FaSdhC1‐H144Y, FaSdhD‐H122Y, FaSdhD‐D133N, FaSdhD‐E166K) also showed a trend of enhanced virulence accompanied by reduced sporulation. It is noteworthy that the enhanced virulence caused by the FaSdhB‐H248Y mutation in this study differs from the previous report (Chen et al. 2021) in the wild‐type background, suggesting that the genetic background plays a critical role in the phenotypic output of resistance mutations. These results indicate that the adaptive effect of resistance mutations is dynamic and is regulated by the composition of the entire SDH complex subunits.

The cross‐resistance analysis further revealed the practical significance of these mutations. The FaSdhB‐H248Y mutant showed resistance to most SDHI fungicides, but remained sensitive to fluopyram. This phenomenon was reported in both *B. cinerea* (Angelini et al. 2014) and *F. pseudograminearum* (Sun et al. 2024), but with different reaction patterns. In *B. cinerea* , different mutation types of SdhB‐H272 (homologous to FaSdhB‐H248) (H272R/Y/V) showed differentiated responses to fluopyram, ranging from high resistance to hypersensitivity, while in *F. pseudograminearum*, the FpSdhB‐H248Y/Q/D mutations all led to increased sensitivity to fluopyram. These findings highlight the functional specificity of histidine at position 248 of SdhB across different species and mutation types. In contrast, other mutants (FaSdhC1‐H144Y/N, FaSdhD‐H122Y, FaSdhD‐D133N, FaSdhD‐E166K) showed resistance to all tested SDHI fungicides, suggesting that such broad‐spectrum resistant types should be vigilantly monitored in production. All resistant mutants remained sensitive to the QoI fungicide pyraclostrobin, providing a theoretical basis for rotation‐based resistance management in the field.

In comparison to previous research, our study elucidates that the wild‐type strain and the *FaSDHC2* knockout strain have exactly the same resistance mechanism to the SDHI fungicides in *F. asiaticum*. We proposed a structural‐level hypothesis: There might be two functional SDH complexes in *F. asiaticum*, namely complex I (SdhA‐SdhB‐SdhC1‐SdhD) and complex II (SdhA‐SdhB‐SdhC2‐SdhD), which coexist within the cell (Figure 7). Their relative abundance determines the baseline sensitivity of the microbial community to SDHI fungicides. To verify this hypothesis, we conducted co‐immunoprecipitation experiments to determine that both FaSdhC1 and FaSdhC2 interacted with the three subunits FaSdhA, FaSdhB and FaSdhD (data not shown). Furthermore, we measured the SDH enzyme activity of *FaSDHC1* and *FaSDHC2* gene knockout mutants, and found that the *FaSDHC2* knockout mutant exhibited decreased enzyme activity and was highly sensitive to SDHI fungicides, which was consistent with the hypothesis expectation: After the deletion of complex II, the strain could only rely on the more sensitive complex I to maintain respiratory function, resulting in an increase in sensitivity to SDHI fungicides. More importantly, although the deletion of *FaSDHC2* led to a decrease in enzyme activity, complex I was still sufficient to maintain the basic growth of the strain and could acquire resistance through other subunit mutations under SDHI fungicides selection pressure, indicating that complex I itself is a fully functional and independently evolving respiratory unit. This provides a key basis for the coexistence of the two complexes at the functional level. On this basis, molecular dynamics simulations further revealed the differences in drug affinity between the two complexes, and the gene knockout experiments verified their non‐redundancy in function, while the screening of resistance mutations confirmed the independent evolutionary ability of complex I. These pieces of evidence collectively form a complete evidence chain from ‘speculating the existence of two complexes’ to ‘verifying functional differences’ and to ‘proving structural independence’, systematically supporting this hypothesis. However, to ultimately clarify the actual assembly form of SDH complex in the cell—whether they exist as two independent tetrameric complexes or dynamically coexist in a larger super‐complex—still depends on direct observation using structural biology techniques.

In summary, this study systematically elucidated the crucial role of SdhC subunit differentiation in regulating the sensitivity of *F. asiaticum* to the SDHI fungicides. We found that FaSdhC1 and FaSdhC2 exerted positive and negative regulatory functions respectively, and confirmed that retaining only FaSdhC1 was more likely to induce resistance. We identified six resistance‐related mutations, four of which were reported for the first time in the *Fusarium* genus, and verified their contributions to the resistance mechanism and adaptive cost at the molecular level. These findings deepened our understanding of the functional differentiation of the SdhC subunit in *F. asiaticum* and the regulatory mechanism of fungicide sensitivity, providing a theoretical basis for the development of new inhibitors targeting FaSdhC2 and the optimization of fungicide application strategies.

## 4 Experimental Procedures

### 4.1 Strains, Medium and Fungicides

The *FaSDHC2* gene deletion mutant ΔFaSDHC2 was obtained from the wild‐type strain 2021 of *F. asiaticum*. All resistant strains were generated through fungicide selection pressure, using the ΔFaSDHC2‐9 strain as the progenitor strain. Additionally, all the site‐directed mutants of *F. asiaticum* were derived from the ΔFaSDHC2‐9 strain.

*Note:* In this study, the names of the genes *FaSDHC1* and *FaSDHC2* were mainly determined based on the results of phylogenetic analysis. The subunit with higher homology to the *SDHC* subunits in common fungi (such as *S. cerevisiae* , *M. oryzae* , *B. cinerea* , *A. fumigatus* , etc.) was named *FaSDHC1*. The other subunit was named *FaSDHC2*. This naming convention is consistent with the phylogenetic and functional nomenclature established by Chen et al. (2024), but it is more scientifically rigorous. In this study, *FaSDHC1* and *FaSDHC2* correspond to *FaSDHC2* and *FaSDHC1* in that article, respectively.

PDA medium (200 g potato, 20 g dextrose, 16 g agar and 1 L water) was used to assay the growth rate of various strains. YBA medium (10 g yeast extraction, 10 g peptone, 20 g anhydrous sodium acetate, 15 g agar and 1 L water) and AEA medium (5 g yeast extraction, 6 g NaNO_3_, 1.5 g KH_2_PO_4_, 0.5 g KCl, 0.25 g MgSO_4_, 16 g agar, 20 mL glycerol and 1 L water) were employed for regular mycelial growth and fungicide sensitivity testing cultures. Boiled mung bean (MBB) medium (30 g boiled mung beans in 1 L water) was used for the determination of sporulation quantity. Tricothecene biosynthesis induction (TBI) medium (30 g sucrose, 1 g KH_2_PO_4_, 0.5 g MgSO_4_·7H_2_O, 0.5 g KCl, 10 mg FeSO_4_·7H_2_O, 800 mg putrescine, 200 μL trace element solution [5 g citric acid, 5 g ZnSO_4_·7H_2_O, 0.25 g CuSO_4_·5H_2_O, 50 mg MnSO_4_·H_2_O, 50 mg H_3_BO_3_ and 50 mg Na_2_MoO_4_·2H_2_O per 100 mL] and 1 L water, pH 6.5) was used to foster the DON biosynthesis.

Technical‐grade fungicides boscalid (active ingredient [a.i.] 98%), fluopyram (a.i. 96%), pydiflumetofen (a.i. 98%), isopyrazam (a.i. 92%), benzovindiflupyr (a.i. 96%) and pyraclostrobin (a.i. 98%) were provided by Taizhou Baili Chemical Co. Ltd., Bayer Co. Ltd., Syngenta Nantong Crop Protection Co. Ltd., Syngenta Nantong Crop Protection Co. Ltd., Syngenta Nantong Crop Protection Co. Ltd. and BASF (China) Co. Ltd., respectively. Additionally, salicylhydroxamic acid (SHAM; Sigma) was used to block alternative oxidation pathways. The fungicides were solubilized in dimethyl sulfoxide at a concentration of 10 mg/mL, while SHAM was prepared in methanol at a concentration of 50 mg/mL, with both solutions stored at 4°C for subsequent use.

### 4.2 Generation of *FaSDHC* Subunits Knockout Mutants in *F. asiaticum* and Sensitivity Tests to SDHI Fungicides Boscalid, Flupyram and Pydiflumetofen

Using the previously established strategy (Song, Li, et al. 2024), we successfully generated knockout mutants designated as ΔFaSDHC1‐6, ΔFaSDHC1‐7, ΔFaSDHC2‐4 and ΔFaSDHC2‐9 for *FaSDHC1* and *FaSDHC2* genes in *F. asiaticum*. The sensitivities of these mutants to the SDHI fungicides boscalid, fluopyram and pydiflumetofen were systematically assessed. The concentration gradients of these fungicides are shown in Table S2. Each concentration was repeated three times, and the experiments were independently performed three times.

### 4.3 Generation of ΔFaSDHC2 Boscalid‐Resistant Mutants

Based on the previous results and methods (Song, Qiu, et al. 2024), the ΔFaSDHC2‐9 strain was designated as the progenitor for the generation of boscalid‐resistant mutants through fungicide taming. A total of 600 fresh mycelial plugs from the ΔFaSDHC2‐9 strain were transferred to YBA plates supplemented with 10 μg/mL boscalid. The sectors with faster growth were then selected for inoculation on plates with higher concentrations of boscalid, 20, 40 and 80 μg/mL. Boscalid‐resistant mutants showed stable growth on plates containing 80 μg/mL of boscalid.

### 4.4 Cross‐Resistance Test

To determine the cross‐resistance of boscalid‐resistant mutants derived from ΔFaSDHC2‐9 to other fungicides with analogous and divergent mechanisms of action, we employed the mycelial growth inhibition method to ascertain the EC_50_ values of boscalid, fluopyram, pydiflumetofen, isopyrazam, benzovindiflupyr and pyraclostrobin. The tests for boscalid, fluopyram, pydiflumetofen, isopyrazam and benzovindiflupyr were executed on YEA medium, while the assessment of pyraclostrobin was conducted on AEA medium augmented with 50 μg/mL of SHAM. The concentration gradients used in this study are detailed in Table S2. Each concentration was replicated three times, and the experiments were independently performed three times.

### 4.5 Biological Characteristics

#### 4.5.1 Mycelial Growth Assay

The linear mycelial growth of both the ΔFaSDHC2‐9 strain and the boscalid‐resistant strains was systematically evaluated. The mycelial plugs were inoculated on PDA plates without fungicides. Following a 72‐h incubation period at 25°C, the colony diameters were measured and growth rates were subsequently calculated. Each treatment for all strains was replicated three times to ensure statistical reliability.

#### 4.5.2 Virulence Assay

Fresh mycelial plugs were cultured in MBB medium at 25°C for 3 days, after which conidial suspensions were collected at a concentration of 5 × 10^5^/mL (Mao et al. 2024). Each conidial suspension (2.5 μL) was inoculated onto wheat coleoptiles, which were cultured in the greenhouse for 10 days. The lengths of lesions on the coleoptiles were recorded. A total of 20 wheat plants were inoculated for each strain, and the experiment was repeated three times.

#### 4.5.3 DON Content Assay

To quantify DON production in the boscalid‐resistant strains, a volume of 10 μL conidial suspension (5 × 10^5^/mL) was added to 30 mL TBI medium, followed by incubation at 28°C in the dark for 7 days. The supernatant was collected, and DON content was assessed utilizing the Vomitoxin (DON) ELISA kit (Wise). The experiment was repeated three times.

### 4.6 Genomic Extraction and Mutation Detection of SDH Subunits

Genomic DNA was extracted from both the parental strain and the resistant mutants by the CTAB method (Mao et al. 2022). The primer pairs P27/P28, P29/P30 and P31/P32 were used to amplify the three subunits of succinate dehydrogenase B, C1 and D in *F. asiaticum* (Table S4). The amplification was performed with 2× Hieff PCR Master Mix (Yeasen) according to the established protocol. Subsequently, the products were sequenced and aligned using BioEdit software (v. 7.2.5.0).

### 4.7 Construction of Site‐Directed Mutations of SDH Subunits in *F. asiaticum*

To validate the accuracy of the identified mutant genotypes, site‐directed mutants were constructed for further analysis. Site‐directed mutagenesis was performed by overlapping extension PCR with minor modifications. In the first step, the first fragments of *FaSDHB*, *FaSDHC1* and *FaSDHD* site‐directed mutation vectors were amplified by P33/P34, P41/PmC1R and P45/PmDR primer pairs, respectively. In the second step, the second fragments of *FaSDHB*, *FaSDHC1* and *FaSDHD* site‐directed mutation vectors were amplified by P35/P36, PmC1F/P42 and PmDF/P46 primer pairs, respectively. In the third step, the primer pairs P37/P38 were used to amplify the *NeoR* (Neomycin Resistance) gene fragment. In the fourth step, the fourth fragments of *FaSDHB*, *FaSDHC1* and *FaSDHD* site‐directed mutation vectors were amplified by primer pairs P39/P40, P43/P44 and P47/P48. The products from these four steps were subsequently fused to generate the final product containing the desired mutations. Confirmation of mutations was performed using primer pairs P49/P50, P51/P52 and P53/P54, as detailed in Table S4.

### 4.8 Screening and Confirmation of Putative Site‐Directed Mutant Transformants

To screen for putative site‐directed mutants, the strain ΔFaSDHC2‐9 was employed as a progenitor for protoplast transformation (Mao et al. 2022). The SRM medium containing 50 μg/mL G418 sulphate was used to select for strains with presumed site‐directed mutations. PCR amplification and subsequent sequencing of the *FaSDHB*, *FaSDHC1* and *FaSDHD* genes were performed using primer pairs P27/P28, P29/P30 and P31/P32 (Table S4). Ultimately, the correct site‐directed mutants were successfully obtained.

### 4.9 Sensitivity Determination of Site‐Directed Mutants to SDHI Fungicides

To evaluate the underlying resistance mechanisms, the sensitivity of the site‐directed mutants ΔFaSDHC2‐SdhB‐H248Y, ΔFaSDHC2‐SdhC1‐H144Y, ΔFaSDHC2‐SdhC1‐H144N, ΔFaSDHC2‐SdhD‐H122Y, ΔFaSDHC2‐SdhD‐D133N and ΔFaSDHC2‐SdhD‐E166K to SDHI fungicides boscalid, fluopyram, pydiflumetofen, isopyrazam and benzovindiflupyr was determined. The concentration gradients for these fungicides are delineated in Table S3.

### 4.10 SDH Enzyme Activity Determination

To determine the SDH activity of the strains, all the strains were cultured in YEPD medium at 25°C, 175 rpm for 36 h. After filtration and dehydration, the mycelia were collected. Then, the SDH activity was determined using the succinate dehydrogenase activity assay kit (Solarbio).

### 4.11 Molecular Dynamics Simulations

The simulated structures of FaSDH Complex I (consisting of FaSdhA, FaSdhB, FaSdhC1 and FaSdhD) and FaSDH Complex II (consisting of FaSdhA, FaSdhB, FaSdhC2 and FaSdhD) were obtained through the AlphaFold Server (https://alphafoldserver.com/). Then, using the GROMACS2025 software package, molecular dynamics simulation analyses of the binding of three SDHI fungicides (boscalid, fluopyram and pydiflumetofen) to FaSDH Complex I and FaSDH Complex II were conducted. The topological structures and coordinate files of the three fungicide molecules were generated using the AMBER 14SB force field and converted to Gromacs' topological structures and coordinate files through pdb2gmx and ACPYPE programs. The topological structures and coordinate files of the complexes and the three fungicide molecules were merged, and immersed in a SPC216 explicit solvent model in a dodecahedron periodic box within a rectangular box. Then, Na^+^ and Cl^−^ counterions were neutralized. The energy minimization was performed using the steepest descent method, followed by position constraint equilibrium in the NVT and NPT ensemble at 298 K and 1.0 bar. The 200‐ns molecular dynamics simulation was conducted using the stepping integrator with a time step of 2 ns. The temperature and pressure were maintained through the modified Berendsen temperature regulator and Parrinello‐Rahman pressure regulator. All bonds involving hydrogen atoms were constrained using the LINCS algorithm. The coordinates and energy of each atom were stored every 10 ns for further analysis. To evaluate the conformational stability and flexibility of the complex in the solvation environment, the root mean square deviation (RMSD) values and the binding free energy Δ*G* were calculated throughout the simulation trajectory.

### 4.12 Data Analysis

Data analysis for the study was conducted using DPS software (DPS v. 7.05). The EC_50_ values were computed through logarithmic conversion of fungicide concentration values and probability conversion of growth inhibition percentages. Fisher's LSD test (*p* = 0.05) was employed to calculate standard errors and assess significant differences among individual EC_50_ values.

## Author Contributions

**Jichang Song:** writing – review and editing, visualization, formal analysis, writing – original draft, data curation, investigation, software. **Jiakai Wang:** investigation, data curation. **Xudong Liu:** software, data curation, methodology. **Yinkai Liu:** investigation, data curation. **Lianyu Bi:** data curation, investigation. **Meixia Li:** investigation, data curation. **Yiqiang Cai:** methodology, investigation. **Mingguo Zhou:** resources, conceptualization. **Yabing Duan:** writing – review and editing, validation, supervision, investigation, project administration, conceptualization, formal analysis, funding acquisition.

## Funding

This work was supported by the National Natural Science Foundation of China (32372578), the Joint Research Program of State Key Laboratory of Agricultural and Forestry Biosecurity (SKLJRP2510) and Graduate Research and Innovation Projects of Jiangsu Province (KYCX25_0985).

## Conflicts of Interest

The authors declare no conflicts of interest.

## Supporting information


**Figure S1:** The vectors construction, verification and growth phenotypes of ΔFaSDHC1 and ΔFaSDHC2 mutants. (a) Construction principle of *FaSDHC1* and *FaSDHC2* gene knockout vectors. (b) Validation of *FaSDHC1* and *FaSDHC2* gene knockout mutants by Southern blot.

**Table S1:** Identification of succinate dehydrogenase subunits in common plant‐pathogenic fungi.

**Table S2:** Fungicide concentrations for the sensitivity tests of ΔFaSDHC1 and ΔFaSDHC2.

**Table S3:** Fungicide concentrations for the sensitivity tests of ΔFaSDHC2 and resistant strains.

**Table S4:** The PCR primers used in this study.

## Data Availability

The data that support the findings of this study are available from the corresponding author upon reasonable request.
