# Supplementary material for: Genetic Differentiation in the SdhC Subunit Confers Intrinsic Resistance to SDHI Fungicides in Fusarium asiaticum
Source: Mol Plant Pathol. 2026 May 5;27(5):e70269. doi: 10.1111/mpp.70269 (PMC13144763; doi:10.1111/mpp.70269)
Supplement: Supplementary file 1 — Figure S1: The vectors construction, verification and growth phenotypes of ΔFaSDHC1 and ΔFaSDHC2 mutants. (a) Construction principle of FaSDHC1 and FaSDHC2 gene knockout vectors. (b) Validation of FaSDHC1 and FaSDHC2 gene knockout mutants by Southern blot. [file MPP-27-e70269-s005.docx]

**
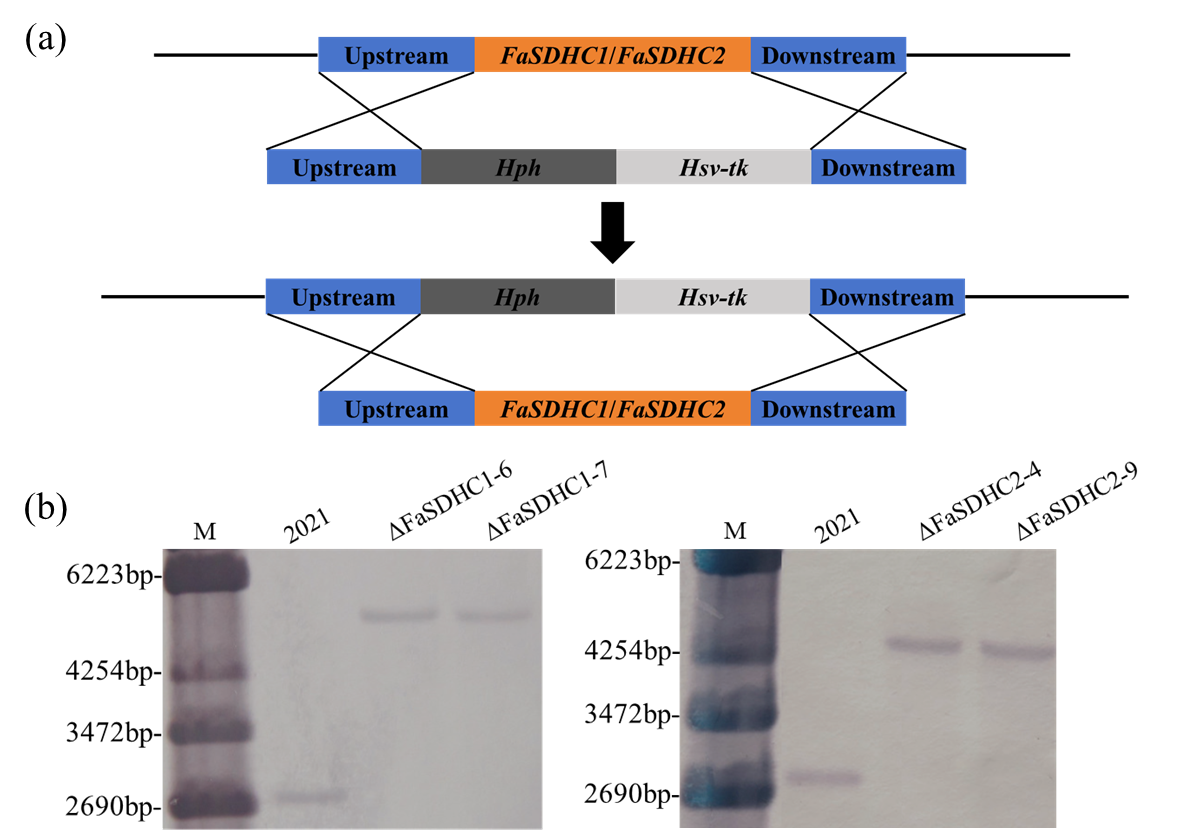
**

**Figure supplement 1** The vectors construction, verification, and growth phenotypes of ΔFaSDHC1 and ΔFaSDHC2 mutants. (a) Construction principle of *FaSDHC1* and *FaSDHC2* gene knockout vectors. (b) Validation of *FaSDHC1* and *FaSDHC2* gene knockout mutants by Southern blot.
