# Supplementary material for: Genetic Differentiation in the SdhC Subunit Confers Intrinsic Resistance to SDHI Fungicides in Fusarium asiaticum
Source: Mol Plant Pathol. 2026 May 5;27(5):e70269. doi: 10.1111/mpp.70269 (PMC13144763; doi:10.1111/mpp.70269)
Supplement: Supplementary file 2 — Table S1: Identification of succinate dehydrogenase subunits in common plant‐pathogenic fungi. [file MPP-27-e70269-s003.docx]

**Table S1 Identification of Succinate Dehydrogenase Subunits in Common Plant Pathogenic Fungi**

| Fungal species | Accession number | | | |
| --- | --- | --- | --- | --- |
|  | SdhA | SdhB | SdhC | SdhD |
| *Saccharomyces cerevisiae* | YJL045W  (NP_012490)  YKL148C  (NP_012774) | YLL041C  (NP_013059) | YKL141W  (NP_012781)  YMR118C  (NP_013836) | YDR178W (NP_010463)  YLR164W  (NP_013265) |
| *Magnaporthe oryzae* | MGG_00168  (XP_003718957) | MGG_00167  (XP_003718958) | MGG_04876  (XP_003712357) | MGG_00666  (XP_003718350) |
| *Fusarium pseudograminearum* | FPSE_04172  (XP_009255565) | FPSE_06201  (XP_009257594) | FPSE_07088  (XP_009258481)  FPSE_05750  (XP_009257143) | FPSE_01262  (XP_009252657) |
| *Fusarium graminearum* | FGSG_13136  (XP_011327122) | FGSG_05610  (XP_011324170) | FGSG_09012  (XP_011328790)  FGSG_01981  (XP_011317844) | FGSG_00743  (XP_011316452) |
| *Fusarium oxysporum* | FOXG_01544  (XP_018234324) | FOXG_09278  (XP_018246409) | FOXG_09077  (XP_018246117)  FOXG_04307  (XP_018238978) | FOXG_01074  (XP_018233601) |
| *Botrytis cinerea* | BCIN_15g01180  (XP_024553221) | BCIN_01g04980  (XP_001548350) | BCIN_02g03080  (XP_001547075) | BCIN_05g04430  (XP_001559774) |
| *Sclerotinia sclerotiorum* | SS1G_07864  (XP_001591238) | SS1G_04384  (XP_001594577) | SS1G_01661  (XP_001597467) | SS1G_06173  (XP_001593251) |
| *Aspergillus fumigatus* | AFUA_3G07810  (XP_754832) | AFUA_5G10370  (XP_753605) | AFUA_5G09680  (XP_753671) | AFUA_1G15590  (XP_752926) |
