# Supplementary material for: Genetic Differentiation in the SdhC Subunit Confers Intrinsic Resistance to SDHI Fungicides in Fusarium asiaticum
Source: Mol Plant Pathol. 2026 May 5;27(5):e70269. doi: 10.1111/mpp.70269 (PMC13144763; doi:10.1111/mpp.70269)
Supplement: Supplementary file 3 — Table S2: Fungicide concentrations for the sensitivity tests of ΔFaSDHC1 and ΔFaSDHC2. [file MPP-27-e70269-s004.docx]

**Table S2 Fungicides concentrations for the sensitivity tests of** **ΔFaSDHC1 and ΔFaSDHC2**

| **Strains** | **Fungicide** | **Concentration (μg/mL)** | | | | |
| --- | --- | --- | --- | --- | --- | --- |
| 2021 | Boscalid | 0.625 | 2.5 | 10 | 40 | 160 |
|  | Fluopyram | 1 | 3 | 9 | 27 | 81 |
|  | Pydiflumetofen | 0.0039 | 0.0156 | 0.0625 | 0.25 | 1 |
| ΔFaSDHC1-6  ΔFaSDHC1-7 | Boscalid | 0.625 | 2.5 | 10 | 40 | 160 |
|  | Fluopyram | 1 | 3 | 9 | 27 | 81 |
|  | Pydiflumetofen | 0.0039 | 0.0156 | 0.0625 | 0.25 | 1 |
| ΔFaSDHC2-4  ΔFaSDHC2-9 | Boscalid | 0.033 | 0.1 | 0.3 | 0.9 | 2.7 |
|  | Fluopyram | 0.002 | 0.01 | 0.05 | 0.25 | 1.25 |
|  | Pydiflumetofen | 0.00097 | 0.0039 | 0.0156 | 0.0625 | 0.25 |
