# Supplementary material for: Genetic Differentiation in the SdhC Subunit Confers Intrinsic Resistance to SDHI Fungicides in Fusarium asiaticum
Source: Mol Plant Pathol. 2026 May 5;27(5):e70269. doi: 10.1111/mpp.70269 (PMC13144763; doi:10.1111/mpp.70269)
Supplement: Supplementary file 4 — Table S3: Fungicide concentrations for the sensitivity tests of ΔFaSDHC2 and resistant strains. [file MPP-27-e70269-s002.docx]

**Table S3 Fungicides concentrations for the sensitivity tests of ΔFaSDHC2 and resistant strains**

| **Strains** | **Fungicide** | **Concentration (μg/mL)** | | | | |  |
| --- | --- | --- | --- | --- | --- | --- | --- |
| ΔFaSDHC2-9 | Boscalid | 0.033 | 0.1 | 0.3 | 0.9 | 2.7 | |
|  | Fluopyram | 0.0025 | 0.01 | 0.05 | 0.25 | 1.25 | |
|  | Pydiflumetofen | 0.00097 | 0.0039 | 0.0156 | 0.0625 | 0.25 | |
|  | Benzovindiflupyr | 0.00625 | 0.025 | 0.1 | 0.4 | 1.6 | |
|  | Isopyrazam | 0.00625 | 0.025 | 0.1 | 0.4 | 1.6 | |
|  | Pyraclostrobin | 0.01 | 0.04 | 0.16 | 0.64 | 2.56 | |
| ΔFaSDHC2-R24  ΔFaSDHC2-R40  ΔFaSDHC2-R41  ΔFaSDHC2-R42  ΔFaSDHC2-R58  ΔFaSDHC2-R67  ΔFaSDHC2-R81  ΔFaSDHC2-R84  ΔFaSDHC2-R112  ΔFaSDHC2-R127  ΔFaSDHC2-R128  ΔFaSDHC2-SdhB-H248Y  ΔFaSDHC2-SdhC1-H144Y  ΔFaSDHC2-SdhC1-H144N  ΔFaSDHC2-SdhD-H122Y  ΔFaSDHC2-SdhD-D133N  ΔFaSDHC2-SdhD-E166K | Boscalid | 5 | 10 | 20 | 40 | 80 | |
|  | Fluopyram | 0.039 | 0.156 | 0.625 | 2.5 | 10 | |
|  | Pydiflumetofen | 0.00097 | 0.0039 | 0.0156 | 0.0625 | 0.25 | |
|  | Benzovindiflupyr | 0.00625 | 0.025 | 0.1 | 0.4 | 1.6 | |
|  | Isopyrazam | 0.00625 | 0.025 | 0.1 | 0.4 | 1.6 | |
|  | Pyraclostrobin | 0.01 | 0.04 | 0.16 | 0.64 | 2.56 | |
