# Supplementary material for: Genetic Differentiation in the SdhC Subunit Confers Intrinsic Resistance to SDHI Fungicides in Fusarium asiaticum
Source: Mol Plant Pathol. 2026 May 5;27(5):e70269. doi: 10.1111/mpp.70269 (PMC13144763; doi:10.1111/mpp.70269)
Supplement: Supplementary file 5 — Table S4: The PCR primers used in this study. [file MPP-27-e70269-s001.docx]

**Table S4 The PCR primers used in this study**

| **Primers** | **Primers Sequence (5'-3')** | **Application** |
| --- | --- | --- |
| P1 | GATCAAGTCAGTGTTAGGCTTTG | Amplify the upstream fragment of *FaSDHC1* gene |
| P2 | TGCTCCTTCAATATCATCTTCTGTTGATGATGGCTTCCTATTCTCGA |  |
| P3 | GAGACAATACCGGAAGGAACCGAGCGGAATAGAAGGAAAACGAG | Amplify the downstream fragment of *FaSDHC1* gene |
| P4 | CTTCGACAGGCTGCTCTG |  |
| P5 | ACAGAAGATGATATTGAAGGAGC | Amplify the *HPH-HSV-tk* gene fragment |
| P6 | GTTCCTTCCGGTATTGTCTC |  |
| P7 | GGCGTCGACCCCTTCAT | Amplify the upstream fragment of *FaSDHC2* gene |
| P8 | GCTCCTTCAATATCATCTTCTGTGCTTGAAATCTAGATAAGCGATCAACTCA |  |
| P9 | GAGACAATACCGGAAGGAACCGAGGTCCATCAGGATTATGC | Amplify the downstream fragment of *FaSDHC2* gene |
| P10 | GCATACGACGCAGGACCT |  |
| P11 | CCTCTTCTCCCACTCGGTAC | Identification the fragment of *FaSDHC1* deletion transformants |
| P12 | ACGTACTTGTCAGCCTGGAT |  |
| P13 | ACTTCACCCGTAACACTGCT | Identification the fragment of *FaSDHC2* deletion transformants |
| P14 | TCCCCAAGTCAAACACCAGG |  |
| P15 | AGTTGTTGACGGGAGGGAAT | Identification the upstream fragment of *FaSDHC1* deletion transformants |
| P16 | CTACTGCTACAAGTGGGGCT |  |
| P17 | TACCCGAGCCGATGACTTAC | Identification the downstream fragment of *FaSDHC1* deletion transformants |
| P18 | GCTGTTCTCGACGACAATCC |  |
| P19 | ATCCAGAAGACCAACAGCGA | Identification the upstream fragment of *FaSDHC2* deletion transformants |
| P20 | CTACTGCTACAAGTGGGGCT |  |
| P21 | GCGACCTGTACAACGTGTTT | Identification the downstream fragment of *FaSDHC2* deletion transformants |
| P22 | TGGAGCAACACCCTGAGATT |  |
| P23 | CGTTGGTGTCTGAGCTGATG | Southern blot validation of *FgSDHC1* deletion mutant |
| P24 | ACCATCTCGACGACACCTTT |  |
| P25 | ATCCAGAAGACCAACAGCGA | Southern blot validation of *FgSDHC2* deletion mutant |
| P26 | GTAGCGGAAGGGGTAGATCC |  |
| P27 | atggctgctctccgatct | Amplify the *FaSDHB* gene fragment |
| P28 | ctagttaccgaaagccatctgc |  |
| P29 | atgctcgctcaacgtgtt | Amplify the *FaSDHC1* gene fragment |
| P30 | ttacaggaaagcaaccagacc |  |
| P31 | atggcctcaattgcgcg | Amplify the *FaSDHD* gene fragment |
| P32 | ttacgccttccagactcgt |  |
| P33 | TTGTTGAGCGCCGATACACC | The first segment of *FaSDHB* site-directed mutagenesis vector was amplified |
| P34 | GGATAGTGTAGCAACGGTACAG |  |
| P35 | CTGTACCGTTGCTACACTATCC | The second segment of *FaSDHB* site-directed mutagenesis vector was amplified |
| P36 | TTCAATATCATCTTCTGTCGACGTGAACGATCAAGAAAATAATATTGCCA |  |
| p37 | GTCGACAGAAGATGATATTGAA | Amplify the *NeoR* gene fragment |
| p38 | TCAGAAGAACTCGTCAAGAAGG |  |
| P39 | CCTTCTTGACGAGTTCTTCTGATTTTGGCTTACCGGTTTCGA | The fourth segment of *FaSDHB* site-directed mutagenesis vector was amplified |
| P40 | GTGCCCTGGCTCGTATAG |  |
| P41 | GGAAGTTATGTGCAACCTCCA | The first segment of *FaSDHC1* site-directed mutagenesis vector was amplified |
| PmC1R-H144Y | GATGGCGTAGTAGACGAATGGG |  |
| PmC1R-H144N | GATGGCGTTGTAGACGAATGGG |  |
| PmC1F-H144Y | TCCCATTCGTCTACTACGCCATC | The second segment of *FaSDHC1* site-directed mutagenesis vector was amplified |
| PmC1F-H144N | TCCCATTCGTCTACAACGCCATC |  |
| P42 | TTCAATATCATCTTCTGTCGACAAGCACATTGTGAGCAAGGAG |  |
| P43 | CCTTCTTGACGAGTTCTTCTGACACGGAAGGCTTCATGGAG | The fourth segment of *FaSDHC1* site-directed mutagenesis vector was amplified |
| P44 | GTGTGTGCGTTATTGTACCTAGT |  |
| P45 | CGAGTCGGTCGGTAGGATC | The first segment of *FaSDHD* site-directed mutagenesis vector was amplified |
| PmDR-H122Y | ATGGGAGTAGAGGAGGAGGAC |  |
| PmDR-D133N | GGGATGTAGTTGATGACGACCT |  |
| PmDR-E166K | CGTTGGTCTTGAACTCGTACAG |  |
| PmDF-H122Y | GTCCTCCTCCTCTACTCCCATA | The second segment of *FaSDHD* site-directed mutagenesis vector was amplified |
| PmDF-D133N | AGGTCGTCATCAACTACATCCC |  |
| PmDF-E166K | GTACGAGTTCAAGACCAACGAC |  |
| P46 | TTCAATATCATCTTCTGTCGACTTAGTACGAAACATAACAATAAATTGAAACAGT |  |
| P47 | CCTTCTTGACGAGTTCTTCTGACACTAAATTCCCTTGTCCTTTTTCATAT | The fourth segment of *FaSDHD* site-directed mutagenesis vector was amplified |
| P48 | GCTTTCGCCTCACCTTTCA |  |
| P49 | TCAGCAACGCCAAAGGC | The *FaSDHB* site-directed mutagenesis vector was sequenced |
| P50 | GTGCCCTGGCTCGTATAG |  |
| P51 | GATCAAGTCAGTGTTAGGCTTTG | The *FaSDHC1* site-directed mutagenesis vector was sequenced |
| P52 | CTTCGACAGGCTGCTCTG |  |
| P53 | ATCGATGGGTAGCAGCC | The *FaSDHD* site-directed mutagenesis vector was sequenced |
| P54 | GCTTTCGCCTCACCTTTCA |  |
